# A stem cell reporter based platform to identify and target drug resistant stem cells in myeloid leukemia

**DOI:** 10.1038/s41467-020-19782-x

**Published:** 2020-11-26

**Authors:** Kyle Spinler, Jeevisha Bajaj, Takahiro Ito, Bryan Zimdahl, Michael Hamilton, Armin Ahmadi, Claire S. Koechlein, Nikki Lytle, Hyog Young Kwon, Ferdous Anower-E-Khuda, Hao Sun, Allen Blevins, Joi Weeks, Marcie Kritzik, Jan Karlseder, Mark H. Ginsberg, Pyong Woo Park, Jeffrey D. Esko, Tannishtha Reya

**Affiliations:** 1 Department of Pharmacology, University of California San Diego School of Medicine, La Jolla, CA USA; 2 Sanford Consortium for Regenerative Medicine, La Jolla, CA USA; 3 Department of Pharmacology and Cancer Biology, Duke University Medical Center, Durham, NC USA; 4 Department of Cellular and Molecular Medicine, University of California San Diego School of Medicine, La Jolla, CA USA; 5 Department of Medicine, University of California San Diego, La Jolla, CA USA; 6 Salk Institute, La Jolla, CA USA; 7 Department of Medicine, Boston Children’s Hospital, Harvard Medical School, Boston, MA USA; 8 Moores Cancer Center, University of California San Diego School of Medicine, La Jolla, CA USA

**Keywords:** Cancer screening, Cancer stem cells, Leukaemia

## Abstract

Intratumoral heterogeneity is a common feature of many myeloid leukemias and a significant reason for treatment failure and relapse. Thus, identifying the cells responsible for residual disease and leukemia re-growth is critical to better understanding how they are regulated. Here, we show that a knock-in reporter mouse for the stem cell gene Musashi 2 (Msi2) allows identification of leukemia stem cells in aggressive myeloid malignancies, and provides a strategy for defining their core dependencies. Specifically, we carry out a high throughput screen using Msi2-reporter blast crisis chronic myeloid leukemia (bcCML) and identify several adhesion molecules that are preferentially expressed in therapy resistant bcCML cells and play a key role in bcCML. In particular, we focus on syndecan-1, whose deletion triggers defects in bcCML growth and propagation and markedly improves survival of transplanted mice. Further, live imaging reveals that the spatiotemporal dynamics of leukemia cells are critically dependent on syndecan signaling, as loss of this signal impairs their localization, migration and dissemination to distant sites. Finally, at a molecular level, syndecan loss directly impairs integrin *β*_7_ function, suggesting that syndecan exerts its influence, at least in part, by coordinating integrin activity in bcCML. These data present a platform for delineating the biological underpinnings of leukemia stem cell function, and highlight the Sdc1-Itgβ7 signaling axis as a key regulatory control point for bcCML growth and dissemination.

## Abstract (web summary)

Identifying leukaemia stem cells (LSC) and defining how they drive tumourigenesis might aid in the treatment of disease. Here, the authors show that a reporter Musashi 2 can serve as a platform to effectively identify leukemic stem cells and it is used to define Syndecan-1 as a dependency for these aggressive, therapy resistant cells.

## Introduction

Over the past several decades, it has become increasingly clear that many cancers are heterogeneous and contain a distinct population of tumor-propagating cells that can also be highly resistant to anticancer therapies^1–5^. Existence of such a therapy-resistant population can explain why many cancers re-emerge after treatment. A key example of this is in chronic myelogenous leukemia (CML), where CML stem cells remain therapy resistant and cannot be eliminated by imatinib (Gleevec), thus resulting in a continued dependence on the drug for a patient’s lifetime. In addition, with the accrual of additional mutations, CML progresses to the aggressive blast crisis phase, which is highly undifferentiated and largely unresponsive to Gleevec^6–10^. Similarly, stem cells in de novo acute myeloid leukemia have been shown to express elevated levels of multidrug resistance genes and exhibit reduced sensitivity to daunorubicin, a standard of care chemotherapeutic in AML^11–13^.

To date, therapy-resistant stem cells in leukemias have predominantly been identified through combinations of surface markers^11,14–17^. While these approaches have been important for prospective isolation of cells and assessment of their role in cancer initiation and propagation, they are limited in their ability to identify and track stem cells in vivo, and cannot be effectively integrated into strategies to screen for dependencies. Thus, the development of efficient ways to identify leukemia stem cells (LSCs) is critically needed to enable real-time detection of therapy-resistant cells, and improve the delineation of their biological underpinnings.

Here we show that a knock-in reporter for the stem cell gene Musashi 2 (Msi2)^18–20^ can serve as a platform to define functional heterogeneity in hematological malignancies, and effectively identify LSCs in both blast crisis chronic myeloid leukemia (bcCML) and de novo AML. By screening for dependencies using this approach, we find several adhesion molecules enriched in therapy-resistant bcCML cells. Among these, syndecan and syndecan-dependent integrin signaling are not only essential for bcCML propagation and lethality but they also control systemic dissemination to distant sites. These data show that Msi reporters represent a key strategy for identifying regulators of LSCs, and highlight adhesive signals as critical players in LSC growth and dissemination.

## Results

### Functional heterogeneity defined by Msi2 in hematologic malignancies

**Fig. 1:**
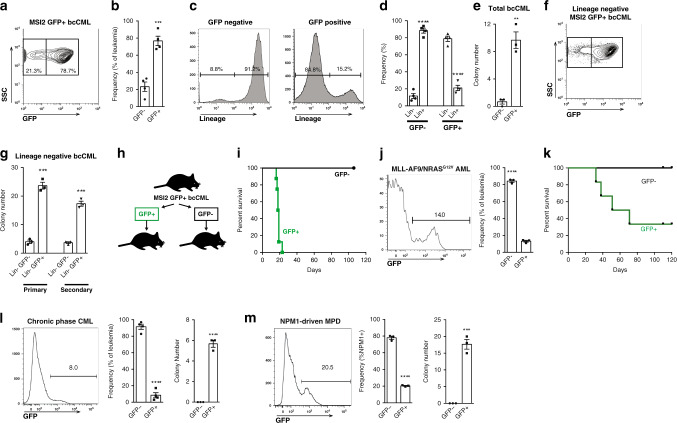
Msi2 marks leukemia stem cells in a mouse model of bcCML. **a** Representative FACS plot shows GFP expression in Msi2-reporter bcCML. **b** Average frequency of GFP^−^negative (GFP^−^) and GFP-positive (GFP^+^) leukemic spleen cells ***P = 0.0005 (*n* = 4 mice). **c** Representative histograms show lineage expression in GFP^−^ and GFP^+^ leukemic spleen cells. **d** The average frequency of lineage-negative (Lin^−^) and lineage-positive (Lin^+^) cells within either the GFP^−^ or GFP^+^ fraction ****P < 0.0001 (*n* = 4 mice per group). **e** Number of colonies generated from GFP^−^ and GFP^+^ bcCML cells. **P = 0.002 (*n* = 3 technical replicates). **f** Representative FACS plot shows GFP expression within the lineage-negative (Lin^−^) fraction of the spleen from a leukemic mouse. **g** Number of colonies generated from Lin^−^ GFP^−^ and Lin^−^ GFP^+^ bcCML cells after primary and secondary plating. ***P = 0.0001 (*n* = 3 technical replicates each). **h** Schematic illustrates an experimental approach to test the ability of established GFP^+^ and GFP^−^ bcCML cells to drive disease development in secondary recipient mice. GFP^+^ or GFP^−^ cells from established bcCML were transplanted into recipients and **i** survival was monitored (*n* = 8 for GFP^+^ and *n* = 10 for GFP^−^). **j** The representative histogram shows GFP expression in leukemic spleen cells from a mouse with MLL-AF9/NRAS^G12V^-driven AML (left panel); frequency of GFP^−^ and GFP^+^ leukemic spleen cells from mice with MLL-AF9/NRAS^G12V^-driven AML ****P < 0.0001 (right panel; *n* = 3 mice per group). **k** Survival of mice transplanted with GFP^−^ and GFP^+^ MLL-AF9/NRAS^G12V^-driven AML (*n* = 6 mice per cohort). **l** The representative histogram shows GFP expression in leukemic spleen cells from a mouse with BCR-ABL-driven CML (left panel); frequency of GFP^−^ and GFP^+^ leukemic spleen cells from mice with BCR-ABL-driven CML ****P < 0.0001 (middle panel, *n* = 4 mice per group); colony formation of Msi2-reporter^+^ and Msi2-reporter^−^ CML ****P < 0.0001 (right panel; *n* = 3 mice). **m** The representative histogram shows GFP expression in leukemic spleen cells from a mouse with NPM1-driven MPD (left panel); frequency of GFP^−^ and GFP^+^ leukemic spleen cells from mice with NPM1-driven MPD ****P < 0.0001 (middle panel, *n* = 3 mice per group); colony formation of Msi2-reporter^+^ and Msi2-reporter^−^ MPD ***P = 0.0003 (right panel; *n* = 3 mice per group). Two-tailed unpaired Student’s *t-*tests were used to determine statistical significance. Source data are provided as a Source Data File.

To test if Msi2-reporter activity can effectively identify LSCs and therapy resistance in myeloid leukemia, we used a BCR-ABL/NUP98-HOXA9-driven model of bcCML^21^. A majority (77%) of bcCML cells derived from Msi2-reporter KLS cells were GFP-positive (Fig. 1a, b), with reporter activity marking the most immature population within bcCML (Lin^−^) (Fig. 1c, d). GFP^+^ cells were also more leukemogenic, as they formed 14.5-fold more colonies than GFP^−^ cells (Fig. 1e), consistent with prior findings on Lin^−^ bcCML cells^22^. Interestingly, Msi2-reporter expression further fractionated Lin^−^ cells into GFP^+^ and GFP^−^ populations (Fig. 1f) with Lin^−^ GFP^+^ cells exhibiting significantly greater colony-forming ability (Fig. 1g), indicating that Msi2-reporter expression marks the more tumorigenic cells within the immature fraction of bcCML. To define whether GFP^+^ leukemia cells are enriched for stem cell activity in vivo, GFP^+^ and GFP^−^ bcCML cells were transplanted and leukemia development monitored. While none of the mice transplanted with GFP^−^ cells developed leukemia, all of the mice transplanted with GFP^+^ cells succumbed to bcCML (Fig. 1h, i), suggesting that the majority of LSC activity in vivo resides within the Msi2-expressing GFP^+^ fraction of the population. We confirmed that the bcCML LSC population (as characterized by Neering et al.^22^) lies exclusively within the Msi2-GFP^+^ population (Supplementary Fig 1a). While only ~0.1% of Msi2-GFP^+^ cells are LSCs, 100% of LSCs were exclusively Msi2-GFP^+^. To determine if the functional demonstration of LSC activity aligned with a molecular LSC signature, we tested whether genes in the previously reported molecular signature of AML LSCs^23^ were also preferentially enriched in Msi2-reporter^+^ bcCML cells. Using RNA Seq analysis of bcCML Lin^−^ cells we assessed expression levels of the mouse homologues of the genes comprising the human leukemic stem cell gene signature (Supplementary Fig 1b). Of these, we selected a subset and directly compared expression in Msi2-reporter^+^ bcCML and Msi2-reporter^−^ bcCML cells by PCR, and found that these genes (*Map3k7, Ppig, Rbpms, Tgif2*, and *Vgll4*) were preferentially higher in Msi2-reporter^+^ bcCML cells relative to the Msi2-reporter^−^ cells (Supplementary Fig. 1c). These data provide a molecular correlate for our finding that Msi2-reporter^+^ cells are functionally enriched in LSC activity.

Interestingly Msi2-reporter activity may mark stem cells in some other hematologic malignancies as well: in an MLL-AF9*/*NRAS^*G12V*^-driven model of AML, all of the lethality associated with the disease was harbored within the GFP^+^ leukemia cells (Fig. 1j, k)^24^. The Msi2 reporter also marked LSCs in chronic myeloid leukemia (Fig. 1l) and in an NPM1-driven model of myeloproliferative disease (Fig. 1m). These data collectively suggest that the Msi2 reporter could serve as a more general strategy to identify stem cells in myeloid malignancies.

### Therapy-resistant population defined by Msi2-GFP reporter activity

**Fig. 2:**
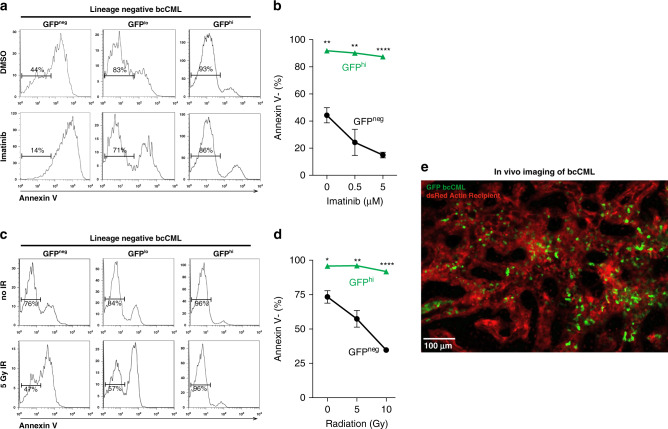
Msi2 marks bcCML cells that are highly therapy resistant. **a**, **b** Msi2-expressing (GFP^hi^) cells are highly resistant to imatinib-induced cell death. **a** Representative histograms show frequency of live (Annexin V-) GFP^neg^, GFP^lo^ and GFP^hi^ established lineage-negative bcCML cells after 7 h of imatinib (5 μM) or DMSO control treatment. **b** The average frequency of live (Annexin V-) GFP^hi^ and GFP^neg^ cells after 7 h of imatinib (500 nM or 5 μM) or DMSO control treatment. **P = 0.001 for 0 μM, **P = 0.002 for 0.5 μM, ****P < 0.0001 for 5 μM (*n* = 4 for each treatment condition and cell type). **c**, **d** Msi2-expressing (GFP^hi^) cells are highly resistant to radiation-induced cell death. **c** Representative histograms show frequency of live (Annexin V-) GFP^neg^, GFP^lo^ and GFP^hi^ established lineage-negative bcCML cells 7 h following radiation (5 Gy). **d** The average frequency of live (Annexin V-) GFP^hi^ and GFP^neg^ cells 7 h following radiation (0, 5, or 10 Gy). *P = 0.01, **P = 0.003, ****P < 0.0001 (*n* = 3 for each treatment condition and cell type). **e** Representative live animal imaging of the calvarium after the establishment of GFP^+^ bcCML; green, donor mouse: actin GFP lin^−^ bcCML, red, recipient mouse: actin dsRed (See also Supplementary Movie 1). Two-tailed unpaired Student’s *t-*tests were used to determine statistical significance. Source data are provided as a Source Data File.

Because imatinib is significantly less effective as CML progresses to bcCML^25,26^, we tested if Msi2-reporter activity also identifies the therapy-resistant cells within bcCML. We specifically analyzed cell survival of GFP^+^ and GFP^−^ cells within Lin^−^ cells to account for any differences that may be solely due to differentiation status. While only 14% of Lin^−^ GFP^−^ cells were viable after imatinib treatment (Fig. 2a, b), GFP^+^ cells were strikingly more resistant with 86% of Lin^−^ GFP^+^ cells remaining viable (Fig. 2a, b). We analyzed this therapy response heterogeneity within the Msi2-reporter^+^ population by analyzing the LSC compartment after exposure to imatinib. We sorted Msi2-GFP^+^ and Msi2-GFP^−^ bcCML cells, incubated with imatinib overnight, and analyzed the bcCML LSC population (Lin^−^CD150^−^Flt3+ Sca1^−^^22^) by flow cytometry. In support of our finding that the LSC population is exclusive to the Msi2-GFP+ fraction, we found that after incubation with imatinib, the LSC content within the Msi2-GFP^+^ fraction was enriched (Supplementary Fig. 1d). This suggests that the LSCs represent the most therapy-resistant cells within the Msi2-GFP^+^ population and endows this population with its chemotherapy resistance and disease propagation capacity. Msi2-reporter activity also marked cells resistant to DNA damaging agents. 96% of the Lin^−^ GFP^+^ cells survived radiation exposure compared to 47% of Lin^−^ GFP^−^ cells (Fig. 2c). Further, while GFP^−^ cells showed increased sensitivity to increasing doses of radiation (0–10 Gy) (Fig. 2d), GFP^+^ cells remained highly resistant, exhibiting 93% viability even at the highest radiation dose used (10 Gy, Fig. 2d). Collectively, these data show that the Msi2-reporter model can be an effective tool to identify therapy-resistant cells in myeloid leukemia.

The fact that Msi2^+^ leukemia cells had a greater capacity to propagate disease and were preferentially able to evade therapy suggested that the reporter might serve as a particularly important tool to identify regulators of these cells. Based on this, we undertook an unbiased high throughput screen to define key dependencies of Msi2^+^ bcCML cells. The screen was focused specifically on cell surface proteins as these provide the main mechanism through which leukemia cells would interact with cells of the microenvironment. In part this decision was based on live imaging showing that leukemia cells are in fact highly interactive with local niches, suggesting that these associations could provide critical cues for their survival and continued propagation (Fig. 2e and Supplementary Movie 1).

### High throughput screen to identify dependencies of Msi2^+^ bcCML cells

**Fig. 3:**
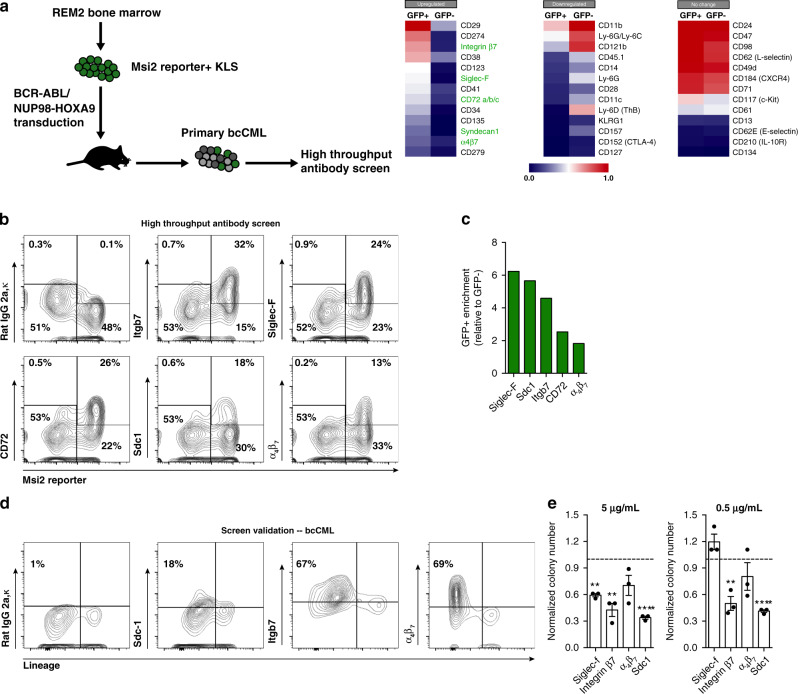
High throughput screen identifies cell surface proteins with functions in Msi2-reporter bcCML cells. **a** Schematic illustrates the experimental approach used for FACS-based high throughput antibody screen to identify surface proteins that are differentially expressed in Msi2^+^ and Msi2^−^ bcCML cells. bcCML was generated from KLS cells derived from Msi2 reporter (REM2) mice. Heat maps display selected genes that are more highly expressed in GFP^+^ bcCML cells (left panel) or GFP^−^ bcCML cells (center panel), or that are unchanged between GFP^+^ and GFP^−^ bcCML cells (right panel) (*n* = 1 technical replicates, representative of two biological replicates for each group). **b** FACS plots generated from high throughput antibody screen show increased expression of the indicated proteins on GFP^+^ bcCML relative to GFP^−^ bcCML. **c** Enrichment of protein expression on GFP^+^ bcCML relative to GFP^−^ bcCML based on high throughput screen (*n* = 1 technical replicates, representative of two biological replicates for each group). **d** Representative FACS plots validating expression of the indicated proteins using individual fluorescently conjugated antibodies in freshly isolated bcCML cells. **e** The relative number of colonies (normalized to IgG control) generated from bcCML cells in the presence of blocking antibodies against the indicated proteins at either 5 μg/mL (left) or 0.5 μg/mL (right). Significance from IgG control: 5 μg/mL, Siglec-f **P = 0.003, Integrin *β*7 **P = 0.004, Sdc1 ****P < 0.0001; 0.5 μg/mL, Integrin β7 **P = 0.004, Sdc1 ****P < 0.0001 (*n* = 3 technical replicates, representative of three biological replicates). Two-tailed unpaired Student’s *t-*tests were used to determine statistical significance. Source data are provided as a Source Data File.

The high throughput screen employed commercially available antibody plates to test a large set of monoclonal antibodies (mAbs) against 176 surface proteins, including 22 IgG isotype control antibodies (Fig. 3a). bcCML cells were incubated in the presence of the antibodies, and subsequently analyzed by flow cytometry to determine expression on GFP^+^ and GFP^−^ cells. Of the 176 mAbs tested, 53 were found to be absent on bcCML cells (<5% of cells positive), and 62 were expressed highly on both GFP^+^ and GFP^−^ cells (>10% of cells positive). We focused specifically on proteins expressed preferentially on GFP^+^ cells, as they could potentially be important for driving differential programs in Msi2^+^ cells. A total of 41 proteins displayed expression greater than twofold higher on GFP^+^ cells compared to GFP^−^ cells. These included proteins that have previously been implicated in CML and bcCML, such as CD34^22^, CD41^27^, CD119^28^, and CD274^29^, serving as an independent validation of this approach. Most importantly, this approach identified multiple surface molecules whose role in myeloid leukemia remains unknown; these included integrin *β*_7_, siglec-F (CD170), CD72, LPAM-1 (integrin *α*_4_*β*_7_) and syndecan-1 (Sdc1, CD138) (Fig. 3a–c).

Given the potential for discovering new programs important for myeloid leukemia, we selected the subset for which blocking antibodies were available and could thus be considered for targeting. Thus we focused on Sdc1, Itgβ7, and *α*_4_*β*_7_, whose expression on bcCML cells was confirmed by subsequent flow cytometry analysis and found to be present at the ratios detected in the screen (Fig. 3d). We also validated the results of our screen using antibodies against a number of proteins that were downregulated (Supplementary Fig. 1e) or unchanged (Supplementary Fig. 1f). To assess their relative function in bcCML, we tracked in vitro colony-forming ability in the presence of blocking antibodies. While inhibition of each had a significant effect on bcCML colony formation, the greatest impact was observed by blocking Sdc1 (clone 281.2, BD Pharmingen), which resulted in a four-fold loss at 0.5 μg/mL and eradication of nearly all colony formation at 5 *μ*g/mL (Fig. 3e). Although based on this we initially prioritized functional analysis of Sdc1, interestingly we later discovered that syndecan and integrin *β*_7_ signaling dependencies were linked.

### Syndecan-1 is necessary for bcCML growth and propagation

**Fig. 4:**
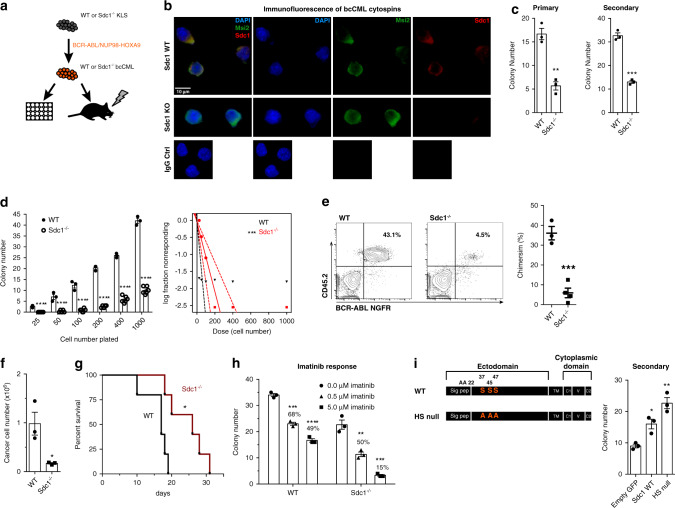
Myeloid leukemia growth and propagation depend on Syndecan-1. **a** Approach to assess the impact of Sdc1 deletion on bcCML. **b** Sdc1 in WT and Sdc1^−/−^ bcCML (blue/DAPI, green/Msi2, red/Sdc1; 63x). **c** Colony formation of WT and Sdc1^−/−^ bcCML. **P = 0.002 primary and ***P = 0.0001 secondary (*n* = 3 technical replicates from four biological replicates). **d** Limiting dilution assay of WT and Sdc1^−/−^ bcCML (LEFT). ****P < 0.0001 (*n* = 3 technical replicates from one biological replicate for WT, *n* = 6 technical replicates from two biological replicates for Sdc1^−/−^). Extreme limiting dilution analysis (RIGHT). Stem cell frequency: WT 1/1, Sdc1^−/−^ 1/94, ***P = 0.0002. **e** Representative chimerism in mice transplanted with WT or Sdc1^−/−^ bcCML. ***P = 0.0007 (*n* = 3 for WT and *n* = 4 for Sdc1^−/−^). **f** Intrafemoral transplant of WT or Sdc1^−/−^ bcCML. *P < 0.03 (*n* = 3 mice for each cohort). **g** Survival curves for WT or Sdc1^−/−^ bcCML. *P = 0.01 (*n* = 5 each cohort). **h** Imatinib response in WT and Sdc1^−/−^ bcCML. Significance from 0 μM: WT ***P = 0.0001, ****P < 0.0001; Sdc1^−/−^ **P = 0.004, ***P = 0.0004 (% is relative to 0 μM for each group, *n* = 3 technical replicates). **i** Ectopic expression of Sdc1/HS-null Sdc1 in Sdc1^−/−^ bcCML. *P = 0.01, **P = 0.002 (*n* = 3 technical replicates from three biological replicates). Two-tailed unpaired Student’s *t-*tests were used to determine statistical significance. Source data are provided as a Source Data File.

Sdc1 is a key mediator of cellular interactions with the microenvironment, can bind and signal in response to a variety of growth factors, and is involved in multiple adhesive processes^30,31^. While syndecan expression has been recently reported on AML patient biopsies^32^, whether it plays a role in regulating myeloid leukemia is not known. To assess the functional role of Sdc1 in leukemia initiation through a definitive genetic model, we used Sdc1 knockout mice developed by disrupting the signaling peptide sequence within the ectodomain, thus preventing mature Sdc1 protein from localizing at the cell surface^33^ (Sdc1^−/−^, Fig. 4a). *Sdc1* deletion was confirmed by PCR (Supplementary Fig. 2a) and immunofluorescence staining (Fig. 4b), and its impact on normal hematopoiesis appeared to be negligible except for a minor increase in Lin^−^ cells (Supplementary Fig. 2b–h). Sdc1^−/−^ KLS cells were infected with BCR-ABL and NUP98-HOXA9, and colony-forming capacity tracked. Compared to control cultures, loss of Sdc1 led to a nearly threefold reduction of bcCML colony formation (Fig. 4c). To assess the impact on LSC function we conducted a limiting dilution series in colony assays (Fig. 4d, Supplementary Fig. 3a). These data show that, at each cell number plated, bcCML colony-forming ability was reduced with Sdc1 loss. Further, with extreme limiting dilution analysis (ELDA) we found that while the stem cell frequency was 1/1 for WT bcCML it was 1/94 for Sdc1^−/−^ bcCML, indicating the Sdc loss triggers a 94 fold drop in stem cell capacity.

Further, transplantation of BCR-ABL/NUP98-HOXA9-infected Sdc1^−/−^ KLS cells resulted in a markedly lower (sevenfold) leukemia burden in vivo 14 days post-transplant (Fig. 4e). This impaired chimerism was not observed at an earlier time point (5 days), suggesting that reduced chimerism is not the result of impaired homing to the bone marrow (Supplementary Fig. 3b). We also observed reduced burden in the femur of recipient mice following intrafemoral transplantation, providing further evidence that Sdc1 loss results in an innate growth defect and not just an inability to home to the bone marrow (Fig. 4f). Most importantly, deletion of Sdc1 led to a 53% gain in survival as measured by median survival (17 days for WT vs. 26 days for Sdc1^−/−^) (Fig. 4g). Since we observed a survival advantage in mice transplanted with Sdc1^−/−^ bcCML, we tested whether Sdc1 loss can sensitize these cells to chemotherapy. Lin^−^ bcCML cells were isolated from mice transplanted with WT or Sdc1^−/−^ bcCML and plated in colony-forming assays in the presence of imatinib. While WT bcCML colony numbers decreased by 50% in the presence of imatinib, Sdc1^−/−^ bcCML colony numbers were reduced by 85% (Fig. 4h). These data suggest that Sdc1 can mediate drug resistance in bcCML LSCs, and that Sdc1 loss can sensitize bcCML cells to imatinib.

To understand the elements of Sdc1 signaling that may be important for its role in bcCML propagation, we tested the ability of wild type or a mutant Sdc1 lacking the heparan sulfate chain attachment sites to rescue the growth of Sdc1^−/−^ bcCML (Fig. 4i). The heparan sulfate chaifns mediate the majority of extracellular interactions of Sdc1, including matrix attachment and signaling of growth factors, whereas the Sdc1 core protein is thought to potentiate inside-out integrin activation^34,35^. Both wild-type Sdc1 and heparan sulfate chain-null Sdc1 were able to rescue colony-forming ability in Sdc1^−/−^ bcCML (Fig. 4i, Supplementary Fig. 3c), suggesting a dependency on the core protein and potentially Sdc1-intergin signaling.

**Fig. 5:**
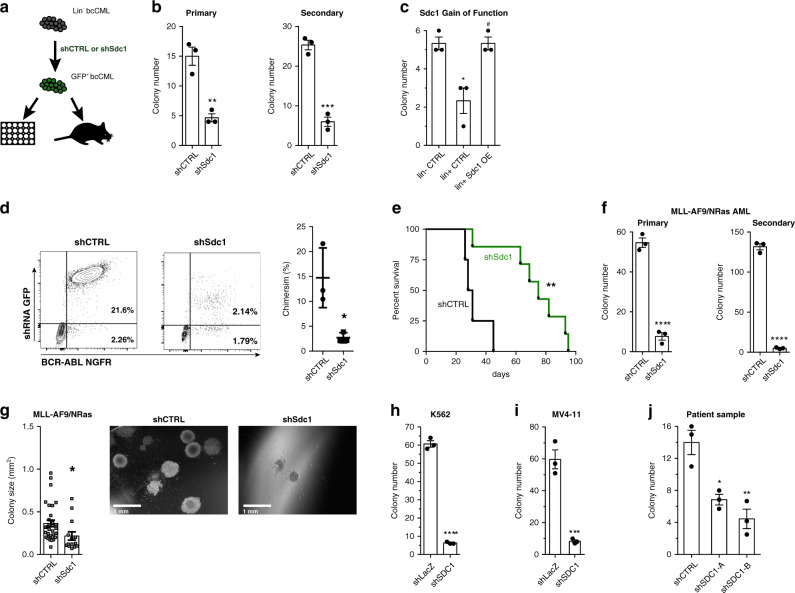
Syndecan-1 loss impairs established bcCML growth in human and mouse models. **a** Approach to assess the impact of Sdc1 loss on established bcCML. **b** Colony formation of shCTRL or shSdc1 bcCML **P = 0.003, ***P = 0.0003 (*n* = 3 technical replicates, representative of three biological replicates). **c** Impact of ectopic Sdc1 expression in Lin^+^ bcCML. *P = 0.02 from Lin^−^CTRL, #*P* = 0.01 from Lin^+^CTRL (*n* = 3 technical replicates). **d** Representative chimerism of shCTRL or shSdc1 bcCML. *n* = 3 mice/group. *P = 0.02. **e** Survival curve of mice transplanted with shCTRL or shSdc1 bcCML. **P = 0.003 (*n* = 4 for shCTRL and *n* = 7 for shSdc1). **f** Colony formation of shCTRL or shSdc1 AML cells. ****P < 0.0001, (*n* = 3 technical replicates). **g** Impact of shSdc1 on AML. *P = 0.03 (*n* = 33 for shCTRL and *n* = 15 for shSdc1). **h** Impact of shSDC1 on K562. ****P < 0.0001 (*n* = 3 technical replicates). **i** Impact of shSDC1on MV4-11. Data normalized to shLacZ control. ***P = 0.001 (*n* = 3 technical replicates from two biological replicates). **j** Impact of shSDC1 on primary patient AML. *P < 0.01, **P = 0.008 (*n* = 3 technical replicates). Two-tailed unpaired Student’s *t-*tests were used to determine statistical significance. Source data are provided as a Source Data File.

To test whether Sdc1 may be important for propagation of established bcCML we assessed the capacity of control and Sdc1-deficient leukemia cells to propagate disease in vitro and in vivo. In vitro, shRNA knockdown of *Sdc1* in established bcCML (Supplementary Fig. 3d) reduced primary colony formation by ~3-fold and secondary colony formation by ~5-fold (Fig. 5a, b). Given our findings that Sdc1 is upregulated on Msi2-reporter^+^ cells (Fig. 3a), the population mainly responsible for driving bcCML growth, we tested whether ectopic overexpression of Sdc1 on Msi2-reporter^−^ bcCML could enhance the growth capacity of these cells. As shown in Fig. 5c, overexpression of Sdc1 in Lin^+^ (Msi2-reporter^−^) bcCML rescued colony growth to levels similar to those of control infected Lin^−^ (Msi2-reporter^+^) bcCML. These findings suggest that Sdc1 expression can drive the acquisition of stem cell characteristics in bcCML. Control and shSdc1 bcCML cells were also transplanted into recipient mice, and disease progression monitored. shSdc1 inhibition resulted in a striking reduction of leukemia burden (~7 fold fewer transplanted cancer cells in peripheral blood) (Fig. 5d) and was significantly less lethal: mice transplanted with wild-type bcCML had a median survival of 31 days, whereas mice harboring shSdc1 bcCML lived 2.5 fold longer with a median survival of 78.5 days (Fig. 5e). Importantly, Sdc1 loss in the MLL-AF9/NRas model of AML (Supplementary Fig. 3e) drastically reduced colony formation in vitro in primary plating by ~5-fold and in secondary plating by more than 10-fold (Fig. 5f, g). These data indicate that Sdc1 is not only critical for bcCML growth, but also for other myeloid malignancies. Importantly, the dependency on syndecan signaling was conserved: thus SDC1 knockdown led to a 12 fold reduction of colony-forming ability in both a human bcCML line and a human AML line (Fig. 5h–i, Supplementary Fig. 3f); expression of *SDC1* and *ITGB7* in these human cell lines was confirmed by qRT-PCR (Supplementary Fig. 3g). Furthermore, knockdown of *SDC1* in primary patient AML led to reduced colony formation as well (Fig. 5j). We should note that other syndecan family members may also contribute to myeloid leukemia, since knockdown of *SDC2*, *SDC3, SDC4* either individually or in combination led to significant growth inhibition (Supplementary Fig. 3h). These data collectively indicate that syndecan signaling is a key dependency of bcCML propagation and lethality, a function at least in part dependent on the core protein and independent of its heparan sulfate chains.

### Loss of Sdc1 affects the spatiotemporal dynamics of bcCML cells

**Fig. 6:**
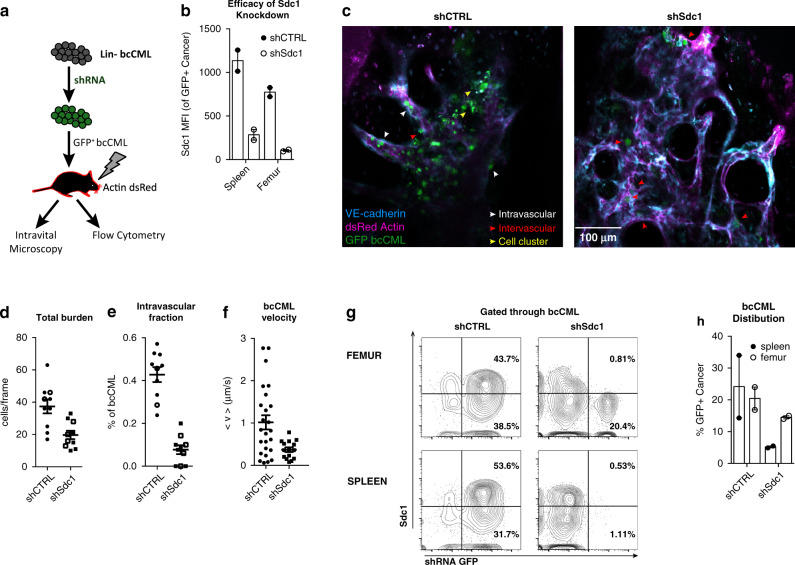
Syndecan-1 controls the spatiotemporal dynamics of bcCML in vivo. **a** Schematic illustrates the experimental approach used to assess the impact of Sdc1 loss on the dynamics of bcCML in vivo. bcCML cells infected with shCTRL or shSdc1were transplanted into irradiated dsRed NOD SCID recipient mice. The calvarium of recipient mice was live imaged 10–15 days post-transplantation, after which the femurs and spleens were analyzed by flow cytometry. **b** Confirmation of Sdc1 knockdown in bcCML cells infected with shSdc1 as quantified as a reduction in Sdc1 MFI by flow cytometry. Significance from shCTRL within a location (*n* = 2 mice per group). **c** Representative live animal imaging of dsRed recipient mice transplanted with shCTRL or shSdc1 bcCML; green = shRNA vector, blue = VE-Cadherin, magenta = Actin dsRed, white arrowhead shows cells within the vasculature, red arrowhead shows cells outside of vasculature within the marrow, yellow arrowhead shows cell clusters (See also Supplementary Movies 2 and 3). **d** Quantification of GFP^+^ bcCML burden from live animal imaging of bone marrow (xyz confocal stacks, *n* = 2 animals per group, open and closed symbols denote separate animals, *n* = 10 fields of view per group). **e** Quantification of GFP^+^ bcCML residing within vasculature determined by live animal imaging (xyz confocal stacks, *n* = 2 animals per group, open and closed symbols denote separate animals, *n* = 10 fields of view per group). **f** The average velocity of GFP^+^ bcCML determined by measuring in vivo cell migration in real time (xyt confocal stacks, *n* = 2 animals per group, ≥3 fields of view per mouse, shCTRL *n* = 25 cells, shSdc1 *n* = 15 cell). **g** Representative flow cytometry plots show the distribution of bcCML in the femur and spleen of mice used in live animal imaging studies in (**b**–**f**). Percentages of shRNA-infected Sdc1+ and Sdc1− bcCML cells are indicated. **h** Quantification of tumor burden in femur and spleen of mice used in the live animal imaging studies (*n* = 2 animals per group). Two-tailed unpaired Student’s *t-*tests were used to determine statistical significance. Source data are provided as a Source Data File.

Because Sdc1 can influence how cells interact with the microenvironment, we used live animal imaging to visually track its impact on the spatiotemporal dynamics of bcCML. GFP-control or GFP-Sdc1 knockdown bcCML cells were transplanted into dsRed recipient mice and imaged (Fig. 6a). Sdc1 knockdown was confirmed by PCR (Supplementary Fig. 4a), as well as by flow cytometry in leukemia cells to ensure that GFP^+^ cells tracked were deficient for Sdc1 expression (Fig. 6b). Imaging revealed that shSdc1 bcCML recipients had nearly 50% fewer donor cells in the bone marrow relative to those receiving control bcCML (Fig. 6c, quantified in 6d), consistent with flow cytometry analysis described above. Unexpectedly, while wild-type cells were present in both the marrow space and in blood vessels at very high frequencies, the decline in Sdc1 knockdown cells was far more pronounced within the blood vessels than within the bone marrow. Specifically, control bcCML cells were more likely to be located within vessels (Fig. 6c, white arrow heads) or clustered together near vascular branching points (Fig. 6c, yellow arrow heads), while shSdc1 cells could most frequently be found as isolated cells, adjacent to but outside of the vasculature (Fig. 6c, red arrow heads), resulting in a fivefold drop of Sdc1 knockdown leukemia cells in blood vessels (Fig. 6e). Further, time-lapse microscopy of individual leukemia cells in the marrow space showed a marked retardation of cell motility. Control leukemia cells traveled at 1.0 ± 0.2 μm/s on average, whereas Sdc1 knockdown leukemia cells traveled at 0.37 ± 0.05 μm/s (Fig. 6f; Supplementary Movies 2 and 3). Interestingly, there was a striking difference in leukemia distribution in the bone marrow and spleen, with loss of Sdc1 leading to a much steeper decline in the spleen (~5-fold reduction compared to control) compared to the bone marrow (~1.3-fold, femur, 2-fold, calvarium) (Fig. 6d, g, h). These data indicate that beyond regulating leukemia burden, Sdc1 expression may also be critical in mediating leukemia cell migration and its systemic dissemination to distant sites.

### Loss of Sdc1 impairs integrin-mediated growth and migration of bcCML cells

**Fig. 7:**
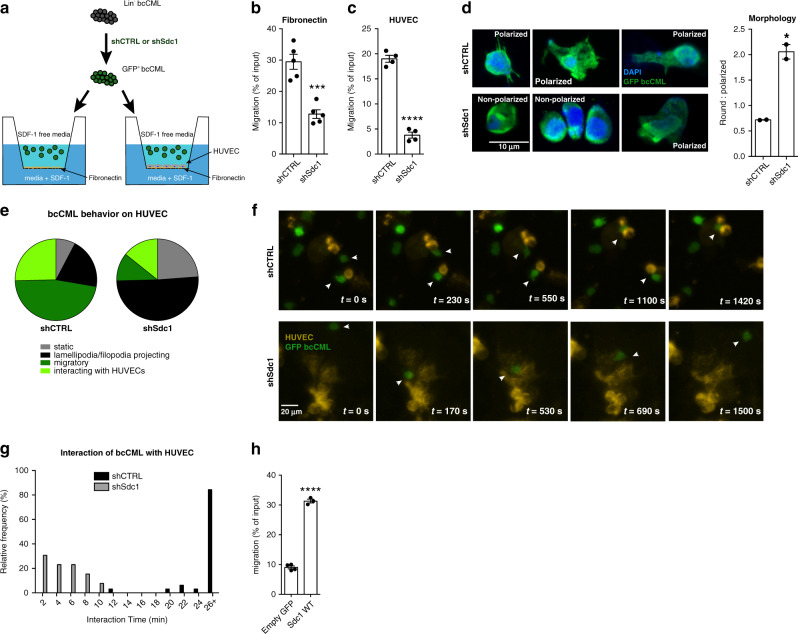
Syndecan-1 mediates cell migration. **a** Schematic of transwell migration assay across either FN or FN+ HUVEC monolayer. **b** SDF-1 induced migration of shCTRL or shSdc1 bcCML cells across fibronectin-coated transwell filters. ***P = 0.0003 (*n* = 3 technical replicates, representative of four biological replicates). **c** SDF-1 induced migration of shCTRL or shSdc1 bcCML cells across HUVEC monolayers seeded on fibronectin-coated transwell filters. ****P < 0.0001 (*n* = 4 technical replicates, representative of two biological replicates). **d** Representative immunofluorescence of shCTRL or shSdc1 bcCML after 4 h incubation on fibronectin-coated transwell filters; green = shRNA vector, blue = DAPI. Quantification of polarized and non-polarized shCTRL or shSdc1 cells. *P = 0.01 (*n* = 3 biological replicates, >30 cells per condition). **e**–**g** Characterization of bcCML behavior when interacting with HUVECs on fibronectin-coated polyacrylamide gels (8 kPa). **e** Cell behaviors are stratified as: completely immobile (gray = static); immobile, but sensing its surroundings by extending lamellipodia and or filopodia (black = lamellipodia/filiopodia projecting); migratory, but not interacting with HUVECs (dark green = migratory); migratory and sustaining prolonged interactions with HUVECs (light green = interacting with HUVECs) (*n* > 10 cells per field of view for four fields across two independent experiments). **f** Representative time-lapse images demonstrating bcCML cell behavior in proximity to HUVECs on fibronectin-coated polyacrylamide gels (See also Supplementary Movies 4 and 5). **g** Duration of interactions between bcCML and HUVECs. (*n* = 33 events for shCTRL and *n* = 13 events for shSdc1). **h** SDF-1 induced migration of Sdc1^−/−^ bcCML cells with empty control or wild-type Sdc1 ectopically expressed across HUVEC monolayers seeded on fibronectin-coated transwell filters. ****P < 0.0001 (*n* = 4 technical replicates). Two-tailed unpaired Student’s *t-*tests were used to determine statistical significance. Source data are provided as a Source Data File.

To understand why Sdc1 depletion influenced the spatiotemporal dynamics of bcCML cells, we tested if Sdc1 knockdown bcCML cells attach and migrate correctly in response to extracellular matrix cues in transwell assays and in context of endothelial cells (Fig. 7a). Sdc1 knockdown in bcCML (Supplementary Fig. 4b) showed a significant reduction in cell migration on extracellular matrix and endothelial monolayers in transwell assays compared to control bcCML (Fig. 7b, c). Cxcr4 levels on Sdc1^−/−^ bcCML cells were not impacted by Sdc1 loss, indicating that the observed migration defect is unlikely to be a result of an inherent reduced capacity to signal through Sdf-1/Cxcr4 (Supplementary Fig. 4c). Imaging of the transwells revealed that a vast majority of control bcCML cells have a polarized morphology; in contrast, shSdc1 cells displayed a significant shift towards a non-polarized morphology (Fig. 7d). The loss in polarization may, at least in part, explain the inability of bcCML lacking Sdc1 to migrate effectively, as directional polarization allows differential attachment to extracellular matrix at the leading edge, an adhesion gradient critical to permitting movement^36^.

Since the live animal imaging studies showed that Sdc1 loss led to fewer bcCML cells in blood vessels, we also modeled transendothelial migration in vitro. Control and Sdc1 knockdown bcCML cells were plated on human endothelial cells (HUVECs) that had been grown on fibronectin-coated polyacrylamide gels with a stiffness comparable to bone marrow (8 kPa). Real-time imaging revealed that bcCML cells lacking Sdc1 were far less motile than control cells (Fig. 7e, f; Supplementary Movie 4). While control cells showed a directed movement towards the endothelial cells and displayed long temporal associations (Fig. 7g), leukemic cells lacking Sdc1 were less motile and non directed in their motion, and remained largely non-interactive with the endothelial cells (Fig. 7e–g; Supplementary Movie 4). Further, ectopic expression of wild-type Sdc1 in Sdc1^−/−^ bcCML (Supplementary Fig. 4d, e) rescued cancer cell migration to near-normal levels (Fig. 7h), indicating a direct role for syndecan in bcCML movement. Collectively, these data highlight a fundamental functional relationship between Sdc1 expression and bcCML migration and localization, and raise the exciting possibility that disabling Sdc1 function could be implemented as a strategy to reduce tumor burden, as well as systemic dissemination.

**Fig. 8:**
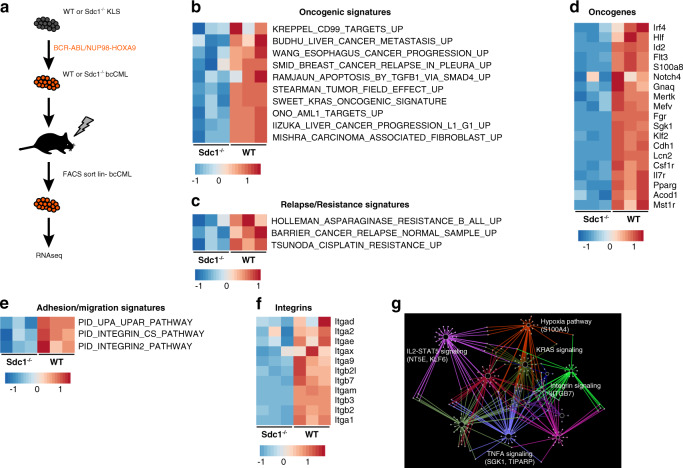
Syndecan-1 loss disrupts growth and migration programs. **a** Schematic of RNAseq workflow. **b** GSEA analysis of WT vs. Sdc1^−/−^ bcCML RNAseq showing impact on oncogenic pathways. **c** GSEA analysis of WT vs. Sdc1^−/−^ bcCML RNAseq showing impact on drug resistance/relapse pathways. **d** Heat map of selected genes falling within the GSEA oncogenic and resistance/relapse pathways. **e** GSEA analysis of WT vs. Sdc1^−/−^ bcCML RNAseq showing impact on adhesion/migration pathways. **f** Heat map of integrin gene expression in Sdc1^−/−^ bcCML falling within the GSEA adhesion/migration pathways. **g** Network analysis of genes downregulated in Sdc1^−/−^ bcCML by RNAseq crossed to a recently published bcCML CRISPR screen.

In an attempt to further understand the impact that Sdc1 loss has on bcCML growth and migration we conducted an RNAseq analysis of WT bcCML versus Sdc1^−/−^ bcCML cells (Fig. 8a). The principal component analysis showed that Sdc1^−/−^ bcCML cells were distinct from WT bcCML cells at a global transcriptional level (Supplementary Fig. 5a) and were defined by the differential expression of hundreds of genes (Supplementary Fig. 5b). Gene set enrichment analysis of the resultant data identified a strong impact on pathways falling within the categories of oncogenic (Fig. 8b) and resistance/relapse (Fig. 8c) that could explain the observed growth defect. Included within these pathways are oncogenic signals such as *Flt3*, *Klf2*, and *Notch4* (Fig. 8d). The migration defect accompanying Sdc1 loss could be explained by the impact on adhesion/migration pathways (Fig. 8e) particularly integrins such as such as *Itgb2, Itgb3, Itga9*, and *Itgax* (Fig. 8f). Interestingly, Itgα2, Itgα9, Itgβ5, and Itgβ7 have been shown to control both growth and migration and may link the observed migration and growth defects. We then crossed the Sdc1^−/−^ bcCML downregulated genes with a recent bcCML CRISPR screen performed in our laboratory^37^. The resulting 111 common genes were then used for Cytoscape enrichment analysis and visualization. This mapping demonstrates the interconnectedness of integrin signaling and oncogenic/survival pathways (Fig. 8g) and the dependence these programs have on Sdc1 in bcCML.

**Fig. 9:**
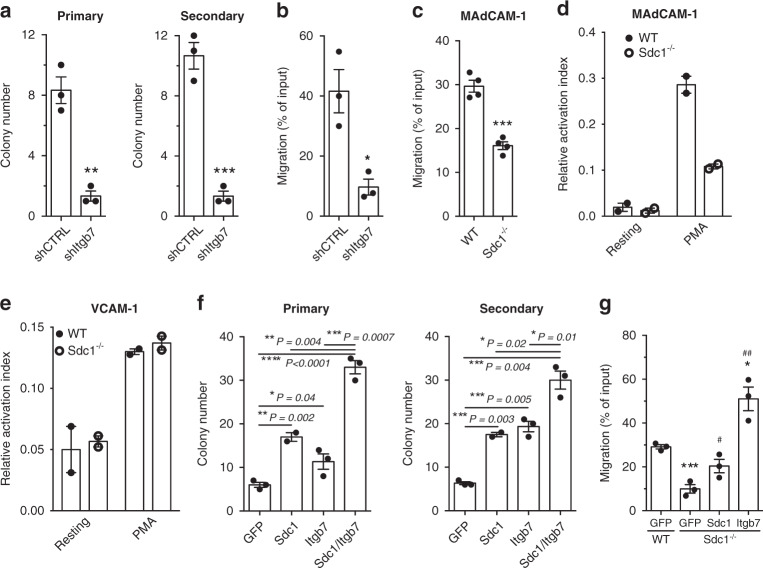
Syndecan-1 loss impairs integrin *β*7 activity. **a** Number of colonies generated from shCTRL or shItgβ7 knockdown of bcCML cells after the primary (left panel) and secondary plating (right panel). **P = 0.001, ***P = 0.0006 (*n* = 3 technical replicates, representative of 3 biological replicates). **b** SDF-1 induced migration of shCTRL or shItgβ7 bcCML cells across HUVEC monolayers seeded on fibronectin-coated transwell filters. *P = 0.01 (*n* = 4 technical replicates, representative of two biological replicates). **c** SDF-1 induced migration of WT or Sdc1^−/−^ bcCML cells across MAdCAM coated transwell filters. ***P = 0.0002 (*n* = 4 technical replicates, representative of two biological replicates). **d** Soluble ligand binding assay with WT and Sdc1^−/−^ bcCML and the Itgβ7 ligand, MAdCAM-1, either resting or stimulated with 200 nM phorbol myristate acetate (PMA). Data are normalized to non-physiological stimulation with 1 mM Mn^2+^ (*n* = 2 technical replicates, representative of two biological replicates). **e** Soluble ligand binding assay with WT and Sdc1^−/−^ bcCML and the Itgβ1 ligand, VCAM-1, either resting or stimulated with 200 nM PMA. Data are normalized to non-physiological stimulation with 1 mM Mn^2+^. (*n* = 2 technical replicates, representative of two biological replicates). **f** Rescue of Sdc1^−/−^ primary and secondary colony formation by ectopic expression of either empty GFP control, wild-type Sdc1, Itgβ7 or Sdc1 + Itgβ7. (*n* = 3 technical replicates, representative of three biological replicates). **g** Rescue of Sdc1^−/−^ migration on FN coated filters by ectopic expression of either empty GFP control, wild-type Sdc1, or Itgβ7. *P = 0.02, ***P = 0.0009, #*P* = 0.04, ##*P* = 0.002. *Significance from WT bcCML +  empty GFP, #significance from Sdc1^−/−^ + empty GFP (*n* = 3 technical replicates, representative of three biological replicates). Two-tailed unpaired Student’s *t-*tests were used to determine statistical significance. Source data are provided as a Source Data File.

To further understand the mechanism by which Sdc1 impacts bcCML growth and migration we focused on integrin *β*_7_ (Itgβ7) as it was identified in our RNAseq analysis and was highly enriched on Msi2^+^ bcCML cells in the high throughput screen (Fig. 3a–c). Its functional inhibition impaired growth of bcCML colony formation (Figs. 3e, 9a and Supplementary Fig. 5c) and bcCML migration on fibronectin-coated filters (Fig. 9b). While the fact that Itgβ7 loss phenocopied Sdc1 loss was consistent with the signals being in a hierarchy, we directly tested this possibility by determining if the absence of Sdc1 function specifically impaired Itgβ7 function. To this end, we repeated the transwell migration experiment using filters coated with the Itgβ7 specific ligand MAdCAM-1, and observed a significant decrease in migration with Sdc1 loss (Fig. 9c). In addition, we performed a soluble ligand binding assay with WT or Sdc1^−/−^ bcCML cells using MAdCAM-1 or VCAM-1, an integrin *α*_4_*β*_1_ ligand. While Sdc1 deletion impaired binding of bcCML cells to MAdCAM-1 by nearly 3-fold (Fig. 9d), binding to the control VCAM-1 was not affected (Fig. 9e). These data confirm that Sdc1 is necessary for the normal function of Itgβ7 and that the coordinated action of Sdc1 and Itgβ7 is a key regulatory element in bcCML. We should note that shSdc1 knockdown did reduce Itgβ7 surface expression by 15 % (Supplementary Fig. 5d); because the majority of integrin surface expression was present even with Sdc1 loss, it may suggest the defects observed are more likely to be due to impaired integrin potentiation. While overexpression of Sdc1 or Itgβ7 could rescue the colony-forming defect observed in Sdc1^−/−^ bcCML to some extent, the two together were more effective in triggering colony growth, suggesting a synergistic mechanism of action (Fig. 9f, Supplementary Fig. 5e). Further, treatment with an Itgβ7 antagonist (firategrast) impaired WT bcCML colony formation but did not impact Sdc1^−/−^ bcCML colony growth (Supplementary Fig. 5f). This finding suggests that in the context of Sdc1 loss, further antagonism of Itgβ7 has no additional impact on colony formation because Sdc1-Itgβ7 signaling is already impaired, while WT bcCML has intact Sdc1-Itgβ7 signaling and is susceptible to Itgβ7 antagonism. Likewise, defects in transwell migration of Sdc1^−/−^ bcCML on FN coated filters could be rescued with wild-type Sdc1 to near-normal levels while rescue of Sdc1^−/−^ bcCML with Itgβ7 increased migration above that of WT control bcCML (Fig. 9g). Finally, while the HS-null Sdc1 mutant could rescue colony formation of Sdc1^−/−^ bcCML (Fig. 4i), the HS-null mutant was unable to rescue the migratory defect (Supplementary Fig. 5g). These data may indicate that the HS chains are critical to “capturing” the cytokine (SDF-1 in this case) or in interacting with the microenvironment to facilitate migration.

## Discussion

Past studies have predominantly used cell surface markers such as CD133 to identify and isolate tumor cells with stem cell-like properties. Although these efforts have enhanced our understanding of tumor stem cell populations, surface markers cannot be effectively used to track cells in vivo and would require complex antibody panels to assess residual disease. In recent years, the ability to identify and track tumor-propagating cells in solid cancers has been greatly improved through the use of reporter mice. For example, in studies of glioblastoma multiforme, Nestin-GFP mice enabled identification of rare Nestin-expressing tumor cells that exhibit many cancer stem cell-like characteristics and are chemo-resistant^38^. Similarly, SOX2-GFP reporter mice were used to identify tumor-propagating cells in skin squamous-cell carcinoma^39^, and an ERG+ 85 reporter has recently been used to identify LSCs within heterogeneous AML samples^40^. Importantly, the Msi2-reporter mouse we developed may be valuable not only for identifying LSCs but for a broader range of aggressive cancers with highly upregulated Msi2 expression, such as hepatocellular carcinoma, glioblastoma and breast cancer^18,41^. The ability to identify therapy-resistant tumor-propagating cells in vivo provides a proof of principle for improving approaches to therapy. For example, following leukemia treatments via cytotoxic and targeted therapies, or radiation prior to allogeneic transplant, image-based detection of therapy-resistant cells could, in the long term, allow areas of residual disease to be identified and targeted with alternative strategies or higher localized doses to improve efficacy and minimize collateral tissue damage.

The screen we performed to identify differentially expressed surface proteins on Msi2^+^ leukemia cells led to the identification of Sdc1 as a key regulator of bcCML growth and propagation. Prior work has implicated Sdc1 in solid cancers such as breast cancer^42,43^ and in multiple myeloma^44–46^. Our discovery that Sdc1 plays a role in bcCML indicates that it may in fact be a core regulatory mechanism for leukemias as well, and suggests that interactions with their niche are a critical source of support for leukemia cells.

A particularly exciting aspect of our studies is the finding that Sdc1 is required for dissemination of bcCML stem cells. Although mechanisms of metastasis in solid tumors have been widely studied, the basis of cell migration in leukemia is generally less understood. Past studies have focused on bone marrow homing, and implicated SDF-1/CXCR4 in B-ALL homing in vivo^47,48^, and CD44^49,50^ in CML and de novo AML homing. Our work is distinct in exploring how leukemia cells migrate away from bone marrow niches, and open ground in our understanding of the molecular basis of bcCML migration by demonstrating that Sdc1 loss leads to a dramatic shift in the spatio-temporal dynamics of bcCML, specifically impairing transit velocity, intravascular frequency and effective colonization of distant sites. Further, the fact that Sdc1 loss impaired adhesion and migration suggests that Sdc1 may control widespread dissemination by facilitating attachment and locomotion on matrix proteins. Syndecans have been shown to have the ability to directly bind matrix proteins such as vitronectin, fibronectin, laminin, and collagens through their heparan sulfate chains^51–53^, and to modulate the ability of cells to interact with the ECM through regulation of integrin activities^54,55^. For example, in multiple myeloma, heparanase trimming of the Sdc1 HS chains allows MMP^−^9-mediated shedding of Sdc1. The shed Sdc1 can then couple VEGFR2 to integrin *α*_4_*β*_1_, resulting in an invasive phenotype^44^. In that context our data show that Itgβ7 is not only enriched on Msi2^+^ leukemic bcCML cells but that Sdc1 directly regulates its function. While Itgβ7 is expressed in lymphomas in addition to AML^56^ its function in hematologic malignancies was unknown. Thus our data provide insight into the role of Itgβ7 in myeloid leukemia, and suggest this could be an exciting avenue of future research. This also provides further support to emerging evidence that Itgβ1 activity, and CD98, which amplifies integrin signaling, can regulate myeloid leukemia^57–59^. The current work adds further insight not only in identifying Itgβ7 as an element in myeloid leukemia but also in defining a role for Sdc1 in coordinating integrin activity in hematologic malignancies. Interestingly, a potential link between Sdc1, Itgβ7, and Msi2 may be found in Msi2’s role as an RNA binding protein. The Msi2 consensus binding sequence (G/AU_1-3_AGU)^60^ has been studied in detail in human, mouse, and drosophila^61^. Analysis of the 3'-UTRs of Sdc1 and Itgβ7 for Msi2 binding sites revealed multiple instances of at least the core element in each transcript (Supplementary Fig. 5h). Therefore, it is possible that Msi2 can directly influence the expression of Sdc1 and Itgβ7. This could be in addition to regulation of the Sdc1 promoter^62^ by the proximal promoter and under the control of members of the Sp1 transcription factor family. While a role for syndecans, integrins, and adhesive signaling has been investigated in the migration and growth of solid cancers^34,54,63^, our data provide a perspective on liquid tumors showing an unexpected importance of adhesion events in leukemia growth and dissemination.

The data discussed here collectively highlight the Sdc1-Itgβ7 signaling axis as a key regulatory control point in sensing microenvironmental cues to regulate bcCML growth and dissemination, and suggest that blocking either Sdc1 or Itgβ7 could weaken such interactions and make leukemic cells more vulnerable to effective targeting. Development of blocking mAbs may be effective in exploiting this vulnerability; certainly the fact that SDC1 inhibition also blocked human bcCML growth provides a strong rationale for further pursuing the possibility of targeting Sdc1 in a clinical setting. An anti-Sdc1 mAb conjugated to chemotherapeutic agents has been developed and is currently in trials for multiple myeloma^45^. Further, a novel CAR T cell targeting Itgβ7 has shown encouraging results in eliminating cancer cells in a murine multiple myeloma model^64^. In this context, our data that Sdc1 and Itgβ7 are highly expressed in bcCML raises the possibility that similar agents could serve as approaches to eradicating LSCs and improving bcCML control.

## Methods

### Mice

All animal experiments were performed according to protocols approved by the University of California San Diego Institutional Animal Care and Use Committee. Mice were bred and maintained in the animal care facilities at the University of California San Diego. The following mice were used: B6-CD45.2 and B6-CD45.1 (Strain: B6.SJL*-Ptprc*^*a*^*Pepc*^*b*^/BoyJ); NSG mice (Strain: NOD.Cg-*Prkdc*^*scid*^ *Il2rg*^*tm1Wjl*^/SzJ); Actin-dsRed mice (also referred as Actb-dsRed. T3 mice, (Strain: NOD.Cg-*Prkdc*^*scid*^Tg(CAG-DsRed*MST)1Nagy/KupwJ). REM2 (Msi2^+/GFP^) reporter mice have been described previously^,65^. Sdc1^−/−^ mice have been previously described^33^. All mice were 8–16 weeks of age. Mice were bred and maintained in the animal care facilities at the University of California, San Diego. All animal experiments were performed according to protocols approved by the University of California, San Diego Institutional Animal Care and Use Committee.

### Generation and analysis of leukemic mice

For *BCR-ABL1/NUP98-HOXA9*-driven blast crisis CML (bcCML), *Mll-AF9*-driven AML, bone marrow-derived KLS cells were isolated and sorted from REM2 mice, CD45.2 B6, or Sdc1^−/−^ mice. All sorted cells were cultured overnight in X-Vivo15 media (Lonza) supplemented with 50 μM 2-mercaptoethanol, 10% (vol/vol) fetal bovine serum, SCF (100 ng/ml, R&D Systems) and TPO (20 ng/ml, R&D Systems). Cells were retrovirally infected with MSCV-BCR-ABL-IRES-NGFR and MSCV-NUP98-HOXA9-IRES-huCD2 to generate bcCML; MSCV-MLL-AF9a-IRES-NGFR and MSCV-NRas^G12V^-IRES-huCD2 to generate MLL-AF9-driven AML. Subsequently, cells were harvested 48 h after infection. For bcCML primary transplants, BCR-ABL^+^/NUP98HOXA9^+^ cells were transplanted retro-orbitally into cohorts of sub-lethally (6 Gy) irradiated CD45.1 mice. For bcCML secondary transplants, BCR-ABL^+^/NUP98-HOXA9^+^ spleen cells recovered from terminally ill primary recipients were sorted and either 5000 GFP^+^ or GFP^−^ leukemia cells were transplanted into sub-lethally (6 Gy) irradiated secondary recipients. For MLL-AF9 primary transplants, MLL-AF9^+^/NRas^+^ cells were transplanted retro-orbitally into cohorts of sub-lethally (6 Gy) irradiated CD45.1 mice. For MLL secondary transplants, MLL-AF9^+^/NRas^+^ spleen cells recovered from terminally ill primary recipients were sorted and either 500 GFP^+^ or GFP^−^ leukemia cells were transplanted into sub-lethally (6 Gy) irradiated secondary recipients. Cell isolation, culture, infection and primary and secondary transplantation assays for *MLL*-driven leukemia were performed as previously described^65^. Disease mice were analyzed as previously described^66^.

### In vitro radiation and imatinib treatment

Bulk blast crisis CML cells recovered from the spleen of terminally ill recipient mice that were initially transplanted with BCR-ABL^+^/NUP98-HOXA9^+^ KLS cells isolated from REM2 mice were either irradiated (0, 5, or 10 Gy) in PBS with glucose and cultured in X-Vivo15 media (Lonza) supplemented with 50 μM 2-mercaptoethanol, 10% (vol/vol) fetal bovine serum, SCF (100 ng/ml, R&D Systems) and TPO (20 ng/ml, R&D Systems) for 7 h or treated with imatinib (0.5 or 5 μM) or control DMSO for 7 h in X-Vivo15 media (Lonza) supplemented with 50 μM 2-mercaptoethanol, 10% (vol/vol) fetal bovine serum, SCF (100 ng/ml, R&D Systems) and TPO (20 ng/ml, R&D Systems). Cells were then washed and stained with antibodies against lineage markers. Apoptosis assays were performed by staining cells with Annexin-V (BD Pharmingen).

### In vitro methylcellulose colony formation assays

For methylcellulose assays performed with blast crisis CML (bcCML) cells, BCR-ABL/NUP98-HOXA9-driven leukemia was generated using KLS cells isolated from B6-CD45.2 or Msi2-reporter mice. Primary bcCML cells were fractionated based on GFP expression (GFP^+^ or GFP^−^) and 250 cells from each fraction were plated in methylcellulose media: Iscove’s modified medium-based methylcellulose medium (Methocult GM M3434, StemCell Technologies). For methylcellulose assays performed with lineage-negative bcCML cells, lineage-negative primary bcCML cells were fractionated based on GFP expression (GFP^+^ and GFP^−^) and 500 cells from each fraction were plated. For serial plating, 500 cells derived from primary colonies were re-plated in fresh methylcellulose media. For serial plating, 5000 cells derived from primary colonies were re-plated. For methylcellulose assays performed with MLL-AF9/NRas AML cells, either 200 MLL-AF9+ /NRas+ GFP^+^ or GFP^−^ spleen cells isolated from leukemic mice were plated in methylcellulose media. For serial plating, 2000 cells derived from primary colonies were re-plated. ImageJ software was used to determine colony size.

### Cell isolation and FACS analysis

Cells were suspended in Hanks’ balanced salt solution (HBSS) (Gibco, Life Technologies) containing 5% (vol/vol) fetal bovine serum and 2 mM EDTA and prepared for FACS analysis and sorting as previously described^67^. The following antibodies were used to define lineage-positive cells: 145-2C11 (CD3ε), GK1.5 (CD4), 53-6.7 (CD8), RB6-8C5 (Ly-6G/Gr1), M1/70 (CD11b/Mac-1), TER119 (Ly-76/TER119), 6B2 (CD45R/B220), and MB19-1 (CD19). Red blood cells were lysed using RBC Lysis Buffer (eBioscience) before staining for lineage markers. The following additional antibodies were used to define HSC populations: 2B8 (CD117/c-kit), D7 (Ly-6A/E/Sca-1), TC15-12F12.2 (CD150), and A2F10 (CD135/Flt3). All antibodies were purchased from BD Pharmingen, eBioscience or BioLegend. The high throughput surface antibody screen was performed per manufacturer’s instruction (BD Lyoplate). In brief, near terminal Msi2-reporter bcCML mice were sacrificed and the spleen was dissociated to produce a single-cell suspension. 500,000 cells were added to each well of a 96 well U-bottom plate. Cells were subsequently incubated with primary, biotin-conjugated secondary, and streptavidin-conjugated fluorescent tertiary antibodies while washing twice between antibody steps. Following tertiary antibody incubation and wash, propidium iodide was added and cells were analyzed using a BD FACSCANTO II equipped with a high throughput sampler. All other experiment analysis and cell sorting were carried out on BD LSRFortessa, FACSCanto and FACSAria II and III machines (all from Becton Dickinson) and data were analyzed with FlowJo software (Tree Star Inc.). Immunofluorescence images were quantified using ImageJ v.1.52a.

### Immunofluorescence staining

Cells were either allowed to settle briefly on poly-L-lysine coated chamber slides (VWR) at 37 °C or cytospun, fixed with 4% paraformaldehyde (USB Corporation), permeabilized with 1X Dako wash buffer (Dako) and blocked with 10–20% normal goat serum (Invitrogen) or donkey serum (Abcam) in 1X Dako wash buffer. Primary antibody incubation was overnight at 4 °C. The following primary antibodies were used: rabbit anti-Msi2 1:200 (Abcam), chicken anti-GFP 1:200 (Abcam) and mouse anti-Sdc1 1:100 (Abcam). Alexa fluor-conjugated secondary antibody incubation was performed for 1 h at room temperature. DAPI (4-6-diamindino-2-phenylindole; Molecular Probes) was used to detect DNA. Images were obtained with a Confocal Leica TCS SP5 II (Leica Microsystems).

### Retroviral constructs and production

MIG-BCR-ABL was provided by Warren Pear and Ann Marie Pendergast and was cloned into the MSCV-IRES-NGFR (or MSCV-IRES-YFP) retroviral vector. MSCV-NUP98-HOXA9-IRES-YFP was provided by Gary Gilliland and was sub-cloned into the MSCV-IRES- huCD2 vector (or MSCV-IRES-NGFR) retroviral vector. MSCV-MLL-AF9-IRES-GFP was provided by Scott Armstrong and was sub-cloned into the MSCV-IRES-NGFR retroviral vector. NRAS^G12V^ cDNA was a gift from Christopher Counter and was cloned into MSCV-IRES-YFP retroviral vector. Wild-type Sdc1 and Sdc1 HS null constructs were subcloned into MSCV-IRES-GFP retroviral vector. Virus was produced in 293T cells transfected using X-tremeGENE HP (Roche) with viral constructs along with VSV-G and gag-pol. Viral supernatants were collected for 3–6 days followed by the ultracentrifugal concentration at 20,000 × *g* for 2 h.

### qRT-PCR analysis

RNA was isolated using RNeasy micro kit (Qiagen) and RNA was converted to cDNA using Superscript III reverse transcriptase (Life Technologies). Quantitative real-time PCRs were performed using an iCycler (BioRad) by mixing cDNAs, iQ SYBRGreen Supermix (BioRad) and gene-specific primers. Gene expression was normalized to the levels of Beta-2 microglobulin (B2M) except for Supplementary Fig. 1c that was normalized to beta-actin (Actb). All primers were designed using NCBI’s Primer-BLAST (https://www.ncbi.nlm.nih.gov/tools/primer-blast/). Primer sequences are listed in Supplementary Data 2.

### Live animal imaging

Live mice were imaged as described previously. In brief, mice were anaesthetized by intraperitoneal injection of ketamine and xylazine (100 and 20 mg/kg respectively). Once unresponsive to pedal reflex, hair was removed using Nair Hair Remover lotion (Church & Dwight Co.) and the scalp was removed to expose the calvarium. Mice were then secured into a mouse/neonatal rat stereotactic holder (Stoelting Co.). The topmost layer of bone was removed with a Dremel 3000 variable speed rotary tool equipped with a 1/16” engraving cutter bit and a 1 cm rubber O-ring was attached to the exposed calvarium (Fine Science Tools Inc.). Images were acquired by Leica LAS AF 2.7.3.9723 software with an upright Leica SP5 2 confocal system using a Leica DM 6000 CFS microscope. Images were acquired using an HCX APO L20× objective with a 1.0 numerical aperture. Resultant videos were adjusted using Adobe Photoshop CS6 version 13.0.

### Transwell chemotaxis assay

Directed cell migration towards SDF1 was analyzed in vitro. Primary murine bcCML was sorted and the Lineage^−^ fraction was transduced with either control shRNA or shSdc1. After 48 h, transduced cells were sorted again for the presence of the shRNA viral construct. Cells were washed, resuspended in IMDM + 0.25% BSA, and plated in the apical chamber of 6.5 mm transwell filters (Costar, pore size 5 μm) that had been coated with fibronectin (Sigma Aldrich) or MAdCAM-1 (R&D Systems) overnight. Six hundred microliters of IMDM + 0.25% BSA + 100 ng/mL SDF-1 (R&D Systems) was added to the basal chamber. Cells were allowed to migrate for 4 h at 37 °C in a 5% CO_2_ incubator. After incubation, migrated cells were collected from the lower chamber and counted using Trypan Blue. For migration across HUVEC monolayers, fibronectin-coated transwells were seeded with HUVECs, and allowed to establish a monolayer. The night before running the experiment, HUVECs were activated with 20 ng/mL TNF-*α* (BD Biosciences). The experiment then proceeded as above.

### Polyacrylamide gel chemotaxis assay

Chemically activated polyacrylamide gels on 18 mm coverslips (8kPa, Matrigen) were coated with 1 μg/mL fibronectin (Sigma Aldrich) before seeding HUVECs. HUVEC monolayer establishment, and activation were as described above. The day of the assay, a chemoattractant gradient was established by incubation of gels with 1 μg/mL SDF-1 for >2 h at 37 °C in a 5% CO_2_ incubator. HUVEC monolayers were stained with CellMask Orange Plasma membrane stain (Invitrogen) before sorted shSdc1 or shCTRL bcCML cells were washed, resuspended in IMDM + 0.25% BSA, and added to the gels. Gels/cells were maintained at 37 °C and 5% CO_2_ while imaging. Images were obtained using a Zeiss LSM-700 confocal microscope.

### WT vs. Sdc1^−/−^ bcCML RNAseq

Lin^−^ leukemic cells were sorted from mice transplanted with BCR-ABL/NUP98-HOXA9 transduced KLS cells. KLS cells were originally sorted from either WT or Sdc1^−/−^ mice to generate WT or knockout leukemia, respectively. Total RNA was isolated using the RNeasy Micro Plus kit (QIAGEN). RNA libraries were generated from 150 ng of RNA using Illumina’s TruSeq Stranded mRNA Sample Prep Kit (Illumina). Libraries were pooled and single-end sequenced (1 × 75) on the Illumina NextSeq 500 using the High output V2 kit (Illumina).

### RNAseq and cell state analysis

Sdc1 KO and WT RNAseq fastq files were processed into transcript-level summaries using *kallisto*^68^. Transcript-level summaries were processed into gene-level summaries and differential gene expression was performed using sleuth with the Wald test^69^. GSEA was performed as previously described^70^. ClueGO was used for gene enrichment analysis of downregulated genes (bias estimator >0.5 and FDR < 0.05) identified between WT and Sdc1 KO mouse cells. PID and Halllmark gene sets were used with medium network specificity, a *p*-value cutoff of <0.05 and a kappa score of 0.4. All other statistical parameters remained on default settings. CluePedia was used to identify genes found within enriched gene sets. All network analyses ran and visualized in Cytoscape 3.7 (Cytoscape, ClueGO/CluePedia).

### Soluble ligand binding assay

5 × 10^6^ cells were washed and resuspended in HBSS containing 0.1% BSA and 1 mM Ca^2+^/Mg^2+^, prior to incubation with integrin ligands (10 μg/ml mouse MAdCAM-1 or 5 μg/ml VCAM-1) for 30 min at 37 °C with or without 200 nM PMA or 1 mM Mn^2+^. Cells were then incubated with AlexFluor647-conjugated anti-human IgG (1:200) for 30 min at 4 °C. Then cells were washed twice before flow cytometry analysis using an Accuri C6 Plus or FACSCalibur (BD Biosciences). Data were analyzed using FlowJo software:

$${\mathrm{Relative}}\,{\mathrm{activation}}\,{\mathrm{index}} = \frac{{\mathrm{MFI} - \mathrm{MFI}_{\mathrm{EDTA}}}}{{\mathrm{MFI}_{\mathrm{Mn}} - \mathrm{MFI}_{\mathrm{EDTA}}}}$$

### Statistics and reproducibility

Statistical analyses were carried out using GraphPad Prism software version 6.0f-h (GraphPad software Inc.). All data are shown as mean ± SEM. Two-tailed unpaired Student’s *t-*tests were used to determine statistical significance. No statistical method was used to predetermine sample size and no data were excluded from the analysis. All experiments were reproducible.

### Reporting summary

Further information on research design is available in the Nature Research Reporting Summary linked to this article.

## Supplementary information

Supplementary Information

Description of Additional Supplementary Files

Supplementary Data 1

Supplementary Data 2

Supplementary Movie 1

Supplementary Movie 2

Supplementary Movie 3

Supplementary Movie 4

Reporting Summary

## Data Availability

Source data are provided with this paper as a separate Source Data file. Antibody information is provided in Supplementary Data 1. Primer information is provided in Supplementary Data 2. Examples of flow cytometry gating strategies are provided as Supplementary Fig. 6. RNAseq data sets have been deposited into the NCBI GEO database under the accession code GSE159148 and are available through the following link. All other data supporting the findings of this study are available within the article and its supplementary information files and from the corresponding author upon reasonable request. Source data are provided with this paper.

## Source data

Source Data
