# Supplementary material for: A stem cell reporter based platform to identify and target drug resistant stem cells in myeloid leukemia
Source: Nat Commun. 2020 Nov 26;11:5998. doi: 10.1038/s41467-020-19782-x (PMC7691523; doi:10.1038/s41467-020-19782-x)
Supplement: Supplementary file 1 — Supplementary Information [file 41467_2020_19782_MOESM1_ESM.pdf]

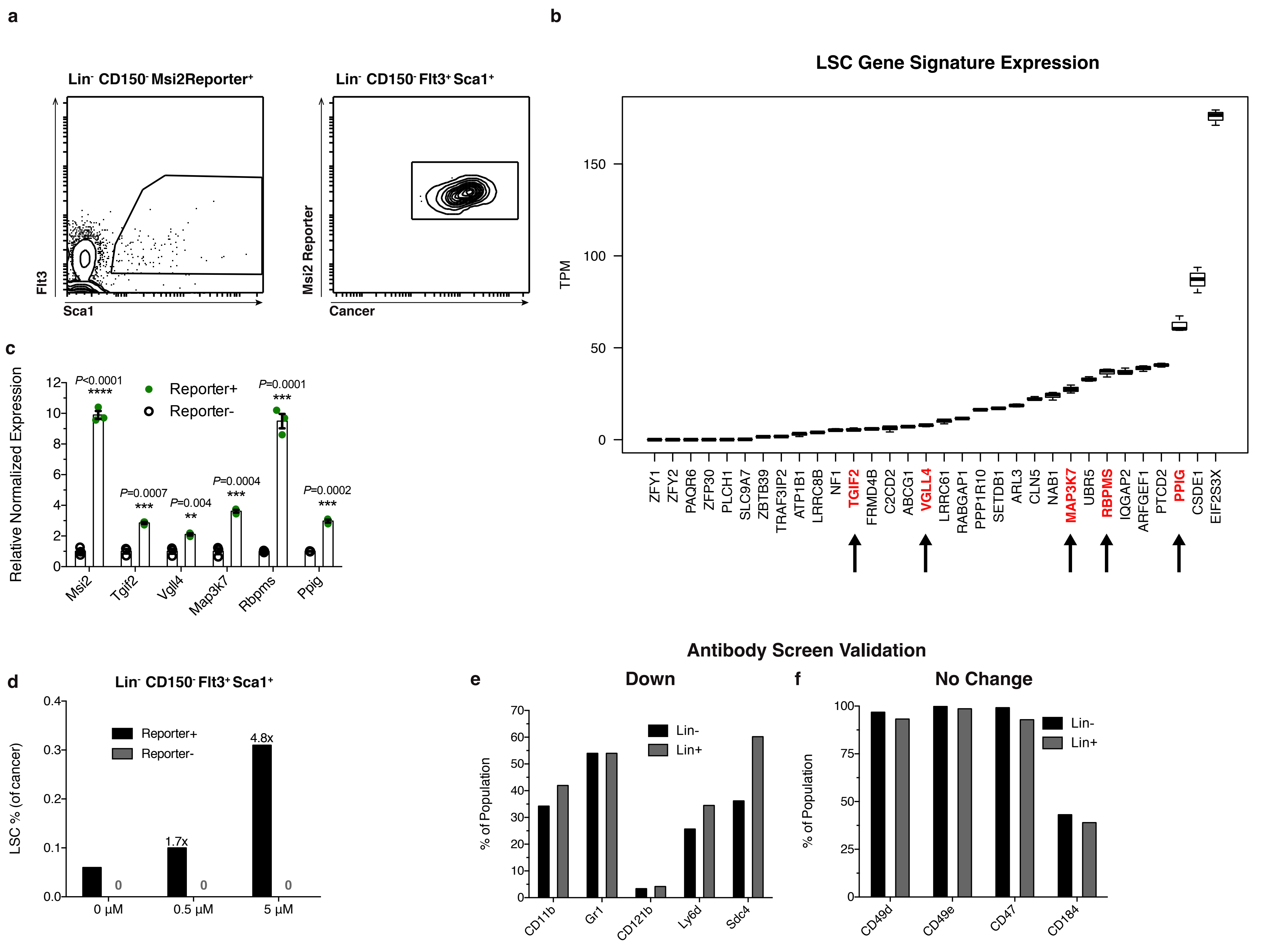

Supplementary Figure 1

Supplementary Figure 1. Confirmation of surface antibody screen

(a) LSC and Msi2 Reporter distribution in bcCML. Lin-CD150-Flt3+Sca1+ within the Msi2 Reporter+ population (**LEFT**). Msi2 Reporter+ within the LSC fraction (**RIGHT**) (representative of 3 biological replicates). (b) Absolute gene expression by RNAseq in Lin- WT bcCML of mouse homologue genes identified as comprising the AML leukemic stem cell signature[21] (*n* = 3 technical replicates, box minima, maxima and center bounds are 25th, 75th, and 50th percentile respectively, arrows denote genes confirmed by qRT-PCR and tested in Supplementary Figure 1c). (c) qRT-PCR of selected LSC genes in Msi2-GFP reporter+ and reporter- bcCML cells sorted from primary transplants (*n*=3 technical replicates, representative of 4 biological replicates). (d) Treatment of Msi2-GFP+ or Msi2-GFP- bcCML cells with imatinib demonstrates the LSC population is exclusive to the Msi2-GFP+ fraction and is resistant to chemotherapy (numbers above the bars indicate the fold change over 0 uM, *n*=1, representative of 2 biological replicates). (e) Flow cytometry confirms the findings of the high throughput antibody screen for proteins downregulated on Msi2-GFP reporter+ bcCML relative to Msi2-GFP reporter- bcCML (*n*=1 technical replicate, representative of 2 biological replicates). (f) Flow cytometry confirms the findings of the high throughput antibody screen for proteins unchanged on Msi2-GFP reporter+ bcCML relative to Msi2-GFP reporter- bcCML (*n*=1 technical replicate, representative of 2 biological replicates). Two-tailed unpaired Student's *t*-tests were used to determine statistical significance.

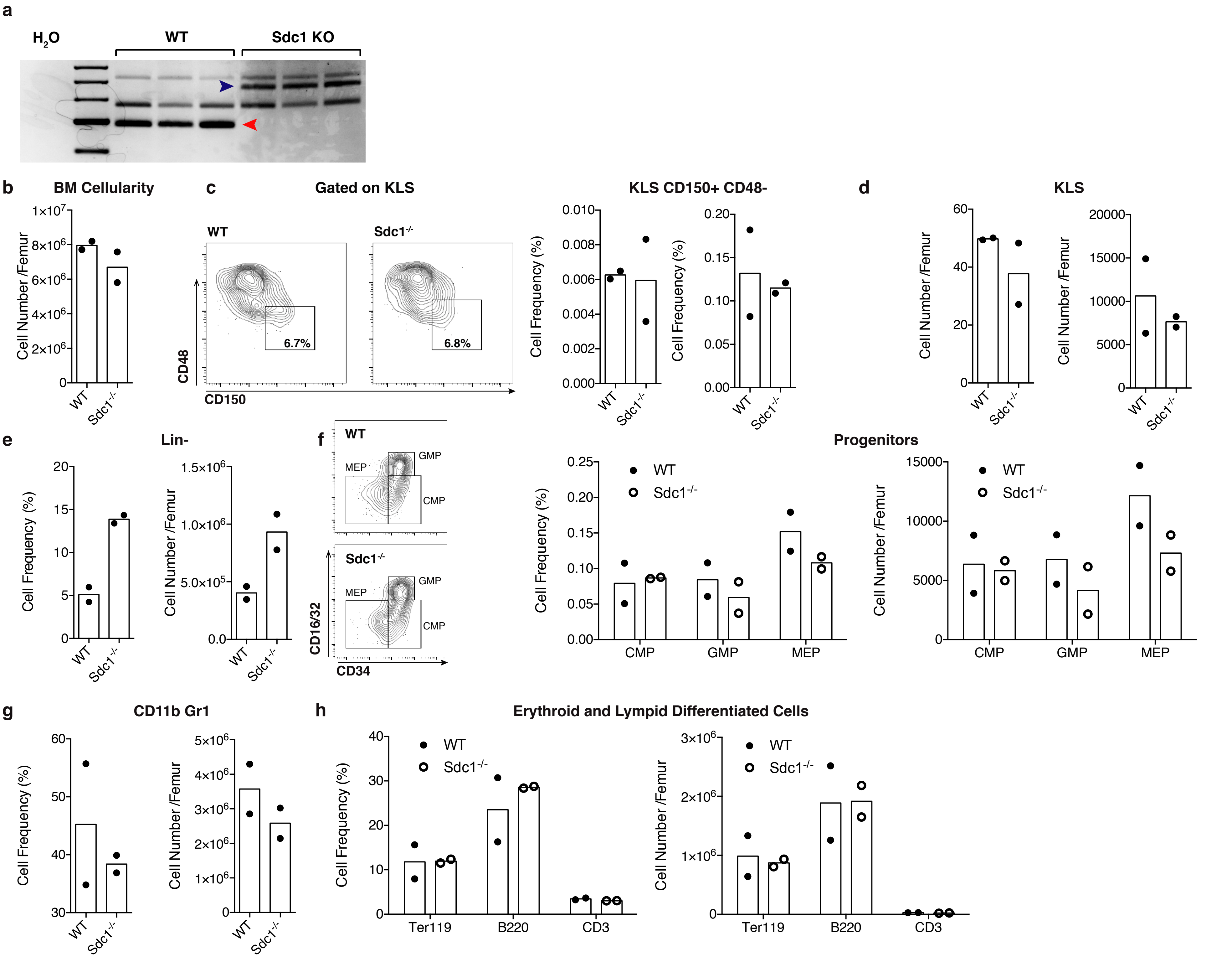

Supplementary Figure 2

Supplementary Figure 2. Analysis of Sdc1 knock out bone marrow

(a) Loss of wildtype Sdc1 in Sdc1 genetic knockout mice confirmed by PCR analysis of genomic DNA. Wildtype band = 230 bp (red arrowhead), knockout band = 450 bp (blue arrowhead), non-specific bands = 300 bp and 500 bp. (b) Total bone marrow cellularity from WT or $Sdc1^{-/-}$ mice (*n*=2 biological replicates). (c) Representative FACS plots (gated on KLS cells), average stem cell (KLS CD150+ CD48-) frequency, and absolute numbers of stem cells in WT or $Sdc1^{-/-}$ mice (*n*=2 biological replicates). (d) Average KLS frequency and absolute cell number in WT or $Sdc1^{-/-}$ mice (*n*=2 biological replicates). (e) Average Lin- frequency and absolute cell number in WT or $Sdc1^{-/-}$ mice (*n*=2 biological replicates). (f) Representative FACS plots show myeloid and erythroid progenitor frequency in WT or $Sdc1^{-/-}$ mice (gated on Lin^{-} $cKit^{+}Sca1^{+}$ $IL7R^{-}$ cells). The average frequency and numbers of progenitors (CMP: $IL7R^{-}KLSCD34^{+}CD16/32^{-}$, GMP: $IL7R^{-}KLSCD34^{+}CD16/32^{+}$, MEP: $IL7R^{-}KLSCD34^{-}$ $CD16/32^{-}$) are shown (*n*=2 biological replicates). (g) Average frequency and absolute number of differentiated myeloid cells in WT or $Sdc1^{-/-}$ mice (*n*=2 biological replicates). (h) Average frequency and absolute number of differentiated erythroid and lymphoid cells in WT or $Sdc1^{-/-}$ mice (*n*=2 biological replicates). Two-tailed unpaired Student's *t*-tests were used to determine statistical significance.

a

responders

dose	WT	Sdc1$^{-/-}$
25	3/3	0/6
50	3/3	2/6
100	3/3	4/6
200	3/3	6/6
500	3/3	6/6
1000	3/3	6/6

confidence intervals

	lower	est.	upper	P
WT	34.1	1.0	1.0	-
Sdc1$^{-/-}$	163.1	94.0	54.2	0.0002

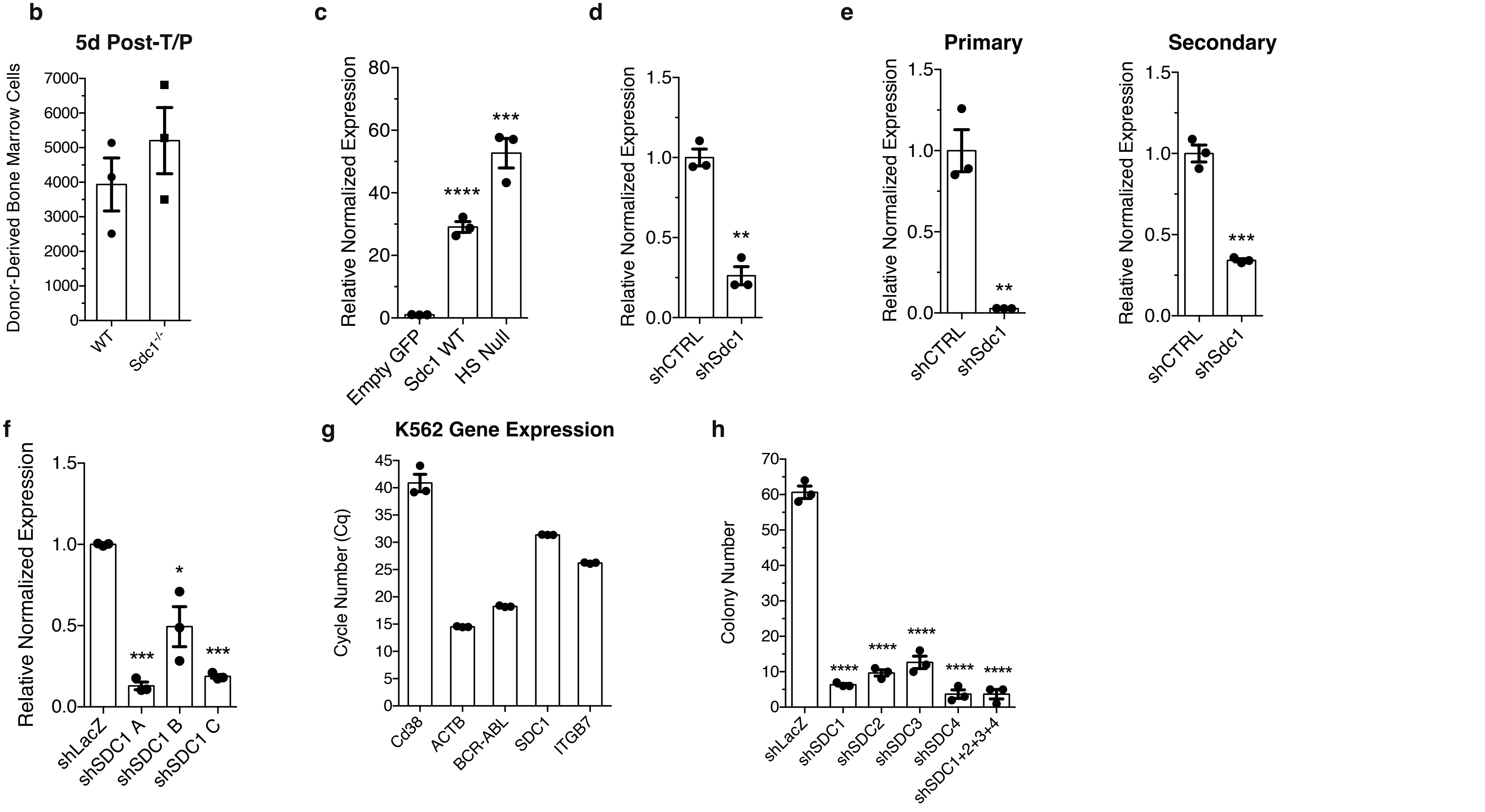

Supplementary Figure 3

Supplementary Figure 3. Analysis of Sdc1 knockdown efficacy and impact in human bcCML cell line

(a) Data from limiting dilution colony assay used in extreme limiting dilution analysis (LEFT, relevant to Fig. 4d). Results from ELDA showing frequency, confidence intervals, and P value for WT vs $Sdc1^{-/-}$bcCML (RIGHT). (b) Chimerism of WT or Sdc1-/- bcCML in murine bone marrow 5 days post-transplant. (*n*=3 biological replicates per cohort). (c) qRT-PCR analysis confirming rescue of Sdc1 expression after infection of $Sdc1^{-/-}$ bcCML cells with wildtype or HS-null Sdc1. Cells used in colony forming assay presented in Fig. 4i. ***P=0.0005, ****P<0.0001 (*n*=3 technical replicates, representative of 3 biological replicates). (d) qRT-PCR analysis confirming knockdown of Sdc1 in established bcCML cells used for colony formation (Fig. 5b) and transplantation (Fig. 5d, e). **P=0.005 (*n*=3 technical replicates, representative of 3 biological replicates). (e) qRT-PCR analysis confirming knockdown of Sdc1 in established MLL-AF9/NRas AML cells used for colony formation experiments (Fig. 5f, 5g). **P=0.002, ***P=0.0006 (*n*=3 technical replicates). (f) Confirmation of SDC1 knockdown in K562 cells. *P=0.04, ***P=0.0001 for both shSDC1 A and C (*n*=3 technical replicates). (g) SDC1 and ITGB7 expression in K562 cells is confirmed by qRT-PCR (*n*=3 technical replicates, mouse Cd38 is used as a negative control, ACTB and BCR-ABL are positive controls). (h) Impact of knockdown of each SDC isoforms impact on K562 colony forming ability. ****P=0.0001 (*n*=3 technical replicates). Two-tailed unpaired Student's *t*-tests were used to determine statistical significance.

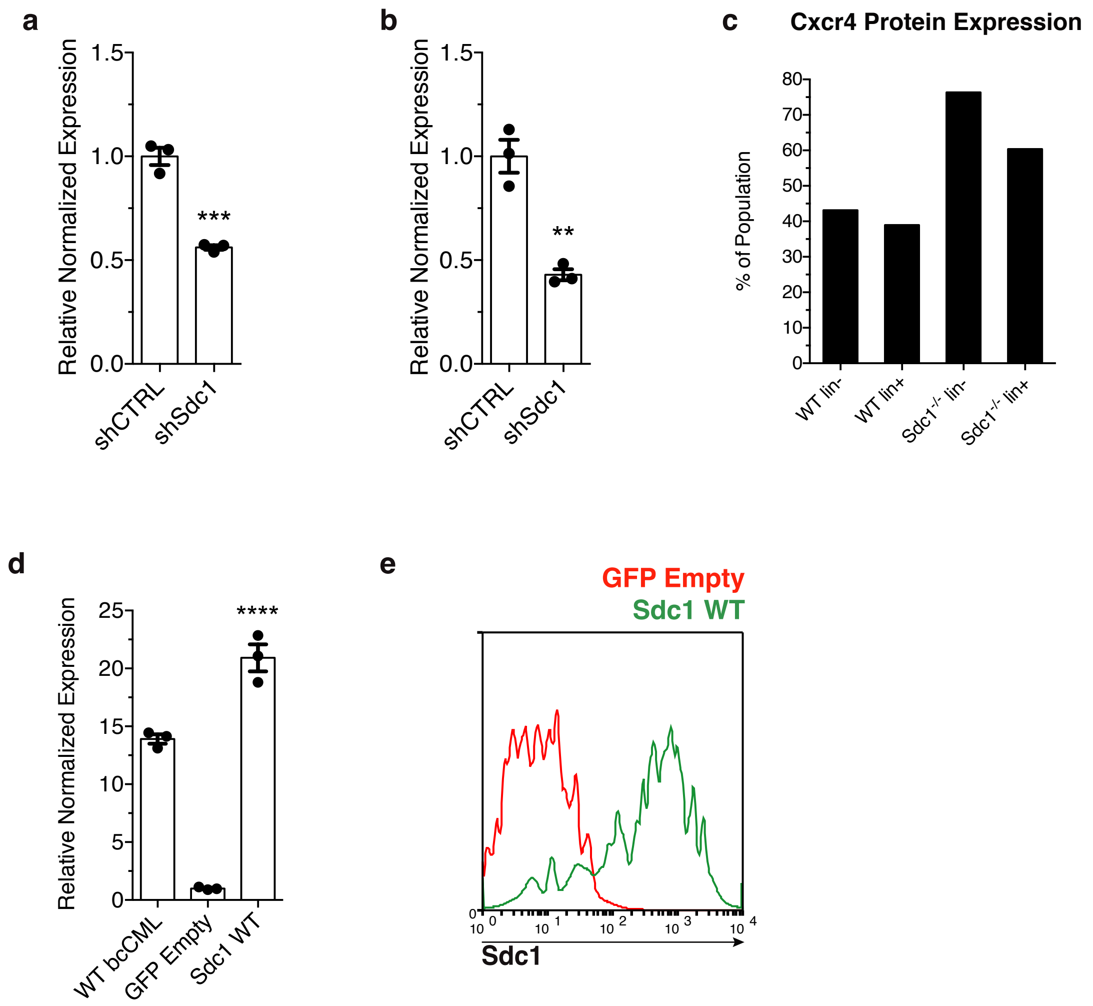

Supplementary Figure 4

Supplementary Figure 4. Sdc1$^{-/-}$ bcCML knockdown and CXCR4 protein expression (a) qRT-PCR analysis confirming knockdown of Sdc1 in established bcCML cells used for *in vivo* imaging experiments (Fig. 6b-h). ****P*=0.0005 (*n*=3 technical replicates, representative of 2 biological replicates). (b) Confirmation of Sdc1 knockdown in bcCML cells by qRT-PCR prior to *in vitro* migration studies (Fig. 7a-g). ***P*=0.002 (*n*=3 technical replicates, representative of 4 biological replicates). (c) CXCR4 protein expression is not reduced in WT vs. Sdc1$^{-/-}$ bcCML either as a percent of positive cells nor is the mean fluorescent intensity. Plot shows % of bcCML cells expressing CXCR4 (*n*=1 WT and 1 Sdc1$^{-/-}$ mouse, representative of 2 biological replicates). (d) Confirmation of Sdc1 rescue by ectopic expression of WT Sdc1 quantified by qRT-PCR. Cells used for *in vitro* migration studies (Fig. 7h). *****P*<0.0001, significance from GFP empty (*n*=3 technical replicates). (e) Flow cytometry of Sdc1$^{-/-}$ bcCML confirms Sdc1 protein expression following ectopic transduction (red = GFP empty, green = WT Sdc1). Two-tailed unpaired Student's *t*-tests were used to determine statistical significance.

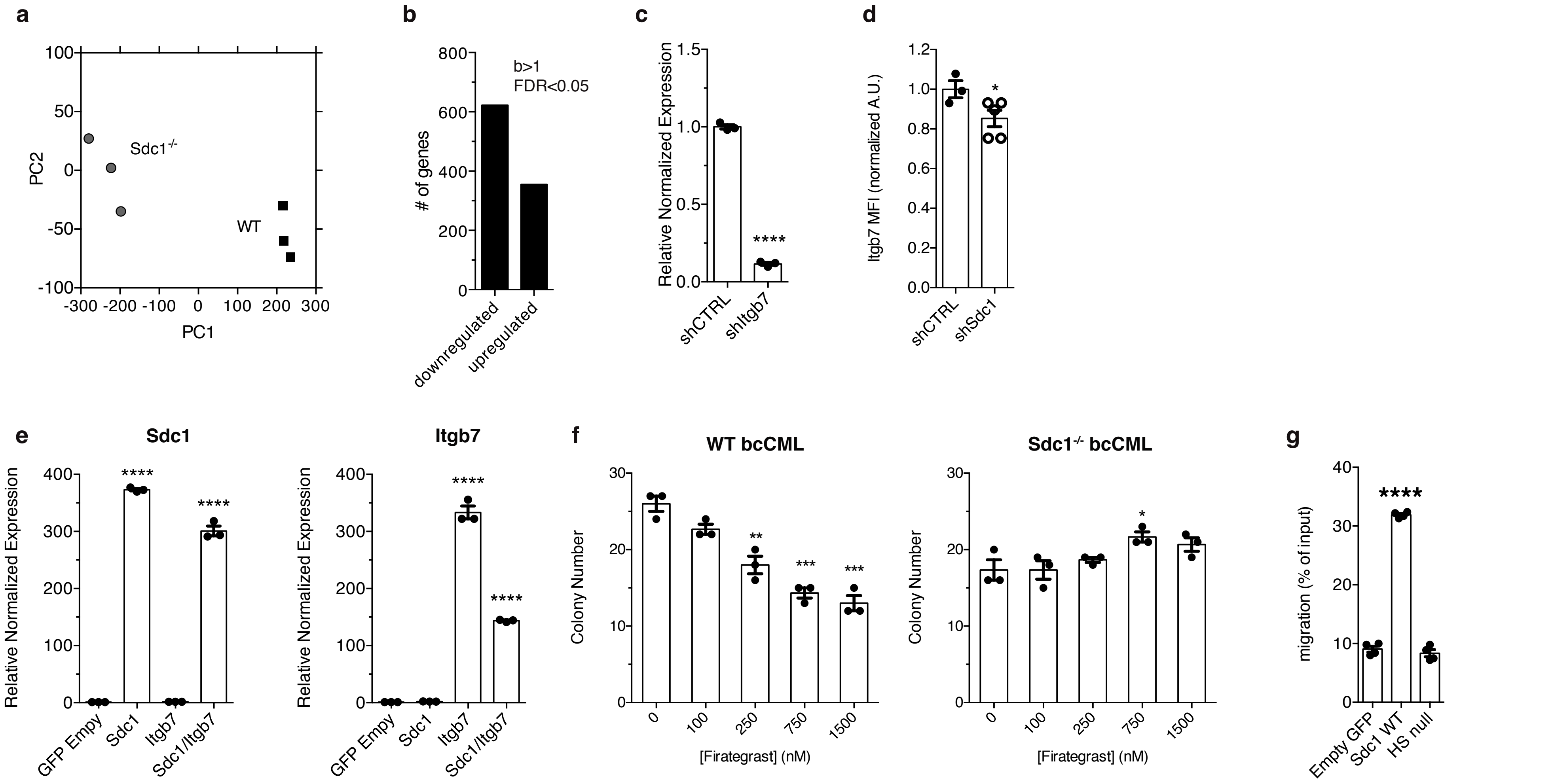

h

Sdc1 3' UTR 1177-3057
NM_011519.2

**UAG**	**UUAG**	**UUAG**
28	3	1
**UAGU**	**UUAGU**	**UUUAGU**
3	1	0
**GUAGU**	**GUUAGU**	**GUUUAGU**
0	0	0
**AUAGU**	**AUUAGU**	**AUUAGU**
1	0	0

Itgb7 3' UTR 2510-2696
NM_013566.2

**UAG**	**UUAG**	**UUAG**
3	1	0
**UAGU**	**UUAGU**	**UUUAGU**
0	0	0
**GUAGU**	**GUUAGU**	**GUUUAGU**
0	0	0
**AUAGU**	**AUUAGU**	**AUUAGU**
0	0	0

Supplementary Figure 5

Supplementary Figure 5. Sdc1$^{-/-}$ bcCML RNAseq and Itgb7 knockdown

(a) Principle component analysis of WT (black square) and Sdc1$^{-/-}$ bcCML (gray circle). (b) Transcripts enriched (by >2-fold) or depleted (by >50%) in Sdc1$^{-/-}$ bcCML relative to WT bcCML with an FDR<0.05. (c) Confirmation of Itgb7 knockdown in bcCML cells by qRT-PCR prior to colony forming assay and *in vitro* migration studies (Fig. 9a, 9b). ****P<0.0001 (n=3 technical replicates, representative of 3 biological replicates). (d) Flow cytometric analysis of Itgb7 protein expression on shCTRL or shSdc1 bcCML. *P=0.05 (n=3 technical replicates for WT, n=5 technical replicates for Sdc1$^{-/-}$). (e) Confirmation of Sdc1 (left) and Itgb7 (right) overexpression in Sdc1$^{-/-}$ bcCML cells by qRT-PCR prior to colony forming assay and *in vitro* migration studies (Fig. 9f, 9g). ****P<0.0001 (n=3 technical replicates, representative of 3 biological replicates). (f) Firategrast antagonism of integrin $\alpha_4\beta_7$ in wildtype (LEFT) and Sdc1$^{-/-}$ bcCML colony formation (RIGHT) (n=3 technical replicates per condition, significance from 0 nM; WT: **P=0.006, ***P=0.0006 for 750 nM, ***P=0.0008 for 1500 nM; Sdc1$^{-/-}$: *P=0.04). (g) Rescue of bcCML migration by ectopic expression of WT or HS null Sdc1 (n=4 technical replicates, representative of 2 biological replicates, ****P<0.001). (h) Frequency of Msi2 consensus binding sequence within the Sdc1 and Itgb7 3' UTR. The mRNA sequence for Sdc1 (LEFT) and Itgb7 (RIGHT) were pulled from NCBI. The 3'UTR for each transcript was scanned for each variation of the Msi2 consensus binding sequence and the occurrences of each are noted in the table. Two-tailed unpaired Student's t-tests were used to determine statistical significance.

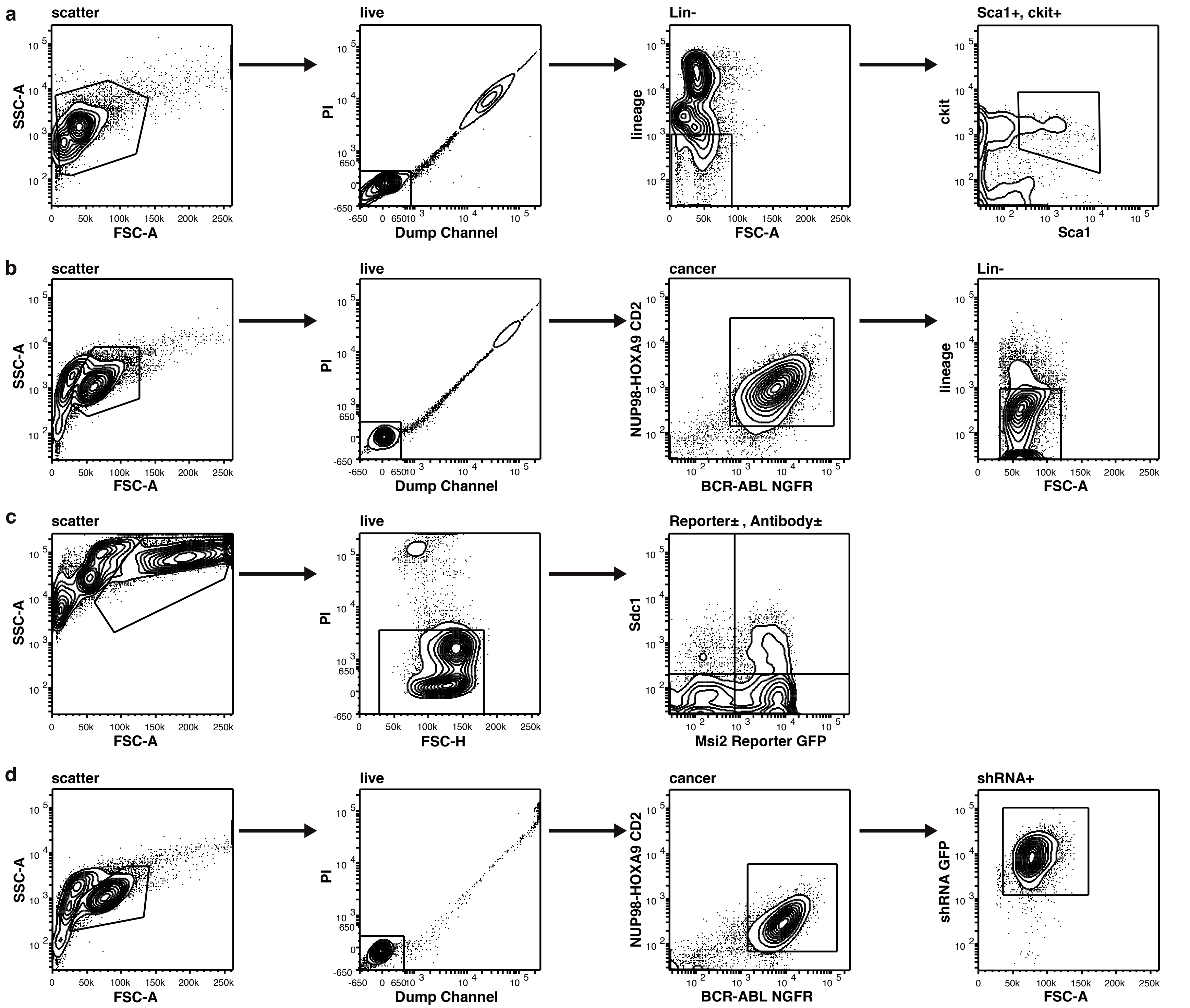

Supplementary Figure 6

Supplementary Figure 6. Example flow cytometry gating strategies used

(a) Gating strategy used for KLS sorting from mouse bone marrow. KLS cells used as starting material for all mouse leukemia models in this paper. (b) Gating used for sorting lin- bcCML from primary transplants. This strategy was used to generate the cell source for all colony forming assays, migration assays, the surface antibody screen, WT vs. Sdc1$^{-/-}$ RNAseq, and upstream of shRNA transduction. (c) Gating scheme used for the surface antibody screen. Lin- bcCML was already sorted prior to running the surface antibody screen. Thus, gating for size and live cells was only necessary before assessing positivity on the MSI2 reporter cells. (d) Gating used for isolating shRNA transduced bcCML. Lin- bcCML cells were previously sorted from primary transplants and infrected with shRNA. After 48hrs cells were sorted again to isolate shRNA+ bcCML cells. This strategy was used for all assays using shRNA.
