# Supplementary material for: A stem cell reporter based platform to identify and target drug resistant stem cells in myeloid leukemia
Source: Nat Commun. 2020 Nov 26;11:5998. doi: 10.1038/s41467-020-19782-x (PMC7691523; doi:10.1038/s41467-020-19782-x)
Supplement: Supplementary file 2 — Description of Additional Supplementary Files [file 41467_2020_19782_MOESM2_ESM.pdf]

Description of Additional Supplementary Files

Supplementary Data 1. List of antibodies
The table shows the list of antibodies for immunofluorescence and flow cytometry used in this study.

Supplementary Data 2. List of primer sequences
The table shows the list of primer sequences used for qRT-PCR and Sdc1$^{-/-}$ mouse genotyping in this study.

Supplementary Movie 1. Real-time imaging of Msi2 reporter bcCML in bone marrow microenvironment.
Imaging of bcCML cells (GFP, green) in bone marrow microenvironment shows long term associations (>4hrs) with local niche cell in dsRed recipient (red). Run time is 4 hours at 24 frames per second.

Supplementary Movie 2. Real-time imaging of CTRL bcCML in bone marrow microenvironment.
Imaging of calvarium of dsRed Actin recipient (magenta) transplanted with Msi2 reporter bcCML (green). Recipient mouse was injected with anti-VE-cadherin (cyan) prior to imaging to illuminate blood vessels. White arrow heads track individual cells of interest to demonstrate representative cellular velocity.

Supplementary Movie 3. Real-time imaging of shSdc1 bcCML in bone marrow microenvironment.
Imaging of calvarium of dsRed Actin recipient (magenta) transplanted with Msi2 reporter bcCML (green). Recipient mouse was injected with anti-VE-cadherin (cyan) prior to imaging to illuminate blood vessels. White arrow heads track individual cells of interest to demonstrate representative cellular velocity.

Supplementa Movie 4. Real-time imaging of shCTRL bcCML cells interacting with HUVECs on fibronectin coated polyacrylamide gel
HUVECs were activated with TNF-α ~24 hours before experiment and labeled with CellTrace Yellow (yellow) just prior to addition of shControl bcCML (green). An SDF-1 gradient was established in the z-plane and cells were kept at 37°C and 5% CO_2 throughout the duration of imaging. Arrow heads track individual cell dynamics and correspond to the behaviors described in Fig. 6e (Red = static, Blue = lamellipodia/filopodia projecting, Yellow = migratory, White = interacting with HUVECs).
