# Supplementary material for: A stem cell reporter based platform to identify and target drug resistant stem cells in myeloid leukemia
Source: Nat Commun. 2020 Nov 26;11:5998. doi: 10.1038/s41467-020-19782-x (PMC7691523; doi:10.1038/s41467-020-19782-x)
Supplement: Supplementary file 9 — Reporting Summary [file 41467_2020_19782_MOESM9_ESM.pdf]

nature research

Corresponding author(s): Tannishtha Reya

Last updated by author(s): Oct 15, 2020

Reporting Summary

Nature Research wishes to improve the reproducibility of the work that we publish. This form provides structure for consistency and transparency in reporting. For further information on Nature Research policies, see Authors & Referees and the Editorial Policy Checklist .

Statistics

For all statistical analyses, confirm that the following items are present in the figure legend, table legend, main text, or Methods section.

n/a	Confirmed	
☐	☒	The exact sample size (*n*) for each experimental group/condition, given as a discrete number and unit of measurement
☐	☒	A statement on whether measurements were taken from distinct samples or whether the same sample was measured repeatedly
☐	☒	The statistical test(s) used AND whether they are one- or two-sided<br>*Only common tests should be described solely by name; describe more complex techniques in the Methods section.*
☒	☐	A description of all covariates tested
☒	☐	A description of any assumptions or corrections, such as tests of normality and adjustment for multiple comparisons
☐	☒	A full description of the statistical parameters including central tendency (e.g. means) or other basic estimates (e.g. regression coefficient) AND variation (e.g. standard deviation) or associated estimates of uncertainty (e.g. confidence intervals)
☐	☒	For null hypothesis testing, the test statistic (e.g. *F*, *t*, *r*) with confidence intervals, effect sizes, degrees of freedom and *P* value noted<br>*Give P values as exact values whenever suitable.*
☒	☐	For Bayesian analysis, information on the choice of priors and Markov chain Monte Carlo settings
☒	☐	For hierarchical and complex designs, identification of the appropriate level for tests and full reporting of outcomes
☒	☐	Estimates of effect sizes (e.g. Cohen's *d*, Pearson's *r*), indicating how they were calculated

Our web collection on statistics for biologists contains articles on many of the points above.

Software and code

Policy information about availability of computer code

Data collection	Flow cytometry and cell sorting was performed on BD FACS Aria III using FACSDiva version 6.1.3 (BD Biosciences).<br>Live animal imaging was performed on a Leica TCS SP5 II using LAS AF version 2.7.3.9723 (Leica).<br>Cell imaging was done on a Zeiss LSM-700 confocal microscope using ZEN 2010B SP1 (Zeiss).<br>qRT-PCR was perfomed on a BioRad CFX96 C100 Thermocycler using BioRad CFX Manager version 3.1 (BioRad).
Data analysis	Statistical analyses were performed using GraphPad Prism 6.<br>Flow cytometry data were analyzed using FlowJo software version 10.5.3.<br>Immunofluorescence images were adjusted using Adobe Photoshop CS6 version 13.0 and images were quantitated using ImageJ (1.52a).<br>RNAseq was analyzed with Cytocape v3.7 and R v3.6 with the following software packages: Kallisto (https://pachterlab.github.io/kallisto/), Sleuth (https://github.com/pachterlab/sleuth), GSVA (https://www.bioconductor.org/packages/release/bioc/html/GSVA.html), limma (https://bioconductor.org/packages/release/bioc/html/limma.html), GSEABase (https://bioconductor.org/packages/release/bioc/html/GSEABase.html), Pheatmap (https://www.rdocumentation.org/packages/pheatmap/versions/1.0.12/topics/pheatmap). All computer code is available upon reasonable request.

For manuscripts utilizing custom algorithms or software that are central to the research but not yet described in published literature, software must be made available to editors/reviewers. We strongly encourage code deposition in a community repository (e.g. GitHub). See the Nature Research guidelines for submitting code & software for further information.

Data

Policy information about availability of data

All manuscripts must include a data availability statement. This statement should provide the following information, where applicable:

- Accession codes, unique identifiers, or web links for publicly available datasets
- A list of figures that have associated raw data
- A description of any restrictions on data availability

Raw data is available in Supplemental Table 3. The RNAseq data that support the findings of this study have been deposited in GenBank (Accession code GSE159148). All other data is available upon request of the corresponding author.

Field-specific reporting

Please select the one below that is the best fit for your research. If you are not sure, read the appropriate sections before making your selection.

[x] Life sciences [ ] Behavioural & social sciences [ ] Ecological, evolutionary & environmental sciences

For a reference copy of the document with all sections, see nature.com/documents/nr-reporting-summary-flat.pdf

Life sciences study design

All studies must disclose on these points even when the disclosure is negative.

Sample size	A minimal number of animals for statistically significant results were used in compliance with the IACUC guidelines. Age and sex matched animals were used for all experiments and all independent experiments. All experiments were repeated as indicated in the legends.
Data exclusions	No data were excluded.
Replication	In vitro experiments were repeated a minimum of 3 times, as stated in the figure legends, and similar results were observed.<br>In vivo experiments were repeated 2-3 times as stated in the legends of each figure. All attempts at replication were successful.
Randomization	Littermate or wild-type control mice from the colony were used for all relevant experiments. Animals enrolled in survival studies were randomly selected to receive either control or knockdown transplants. Mice were randomly assigned for bone marrow/spleen analysis and allowed to continue for survival studies for all relevant experiments.
Blinding	Investigators were blinded for colony forming assays and to patient sample details. The investigators were not blinded during outcome assessment for other experiments since data acquisition and analysis were done using indicated software.

Reporting for specific materials, systems and methods

We require information from authors about some types of materials, experimental systems and methods used in many studies. Here, indicate whether each material, system or method listed is relevant to your study. If you are not sure if a list item applies to your research, read the appropriate section before selecting a response.

Materials & experimental systems

n/a	Involved in the study
[ ]	[x] Antibodies
[ ]	[x] Eukaryotic cell lines
[x]	[ ] Palaeontology
[ ]	[x] Animals and other organisms
[x]	[ ] Human research participants
[x]	[ ] Clinical data

Methods

n/a	Involved in the study
[x]	[ ] ChIP-seq
[ ]	[x] Flow cytometry
[x]	[ ] MRI-based neuroimaging

Antibodies

Antibodies used	A detailed list of antibodies used for immunofluorescence and flow cytometry (including vendor, catalog number, and dilution) is found in Supplementary Table 1.
Validation	Pre-validated antibodies available commercially were used for the study. All antibodies have been tested by the manufacturer for the relevant application on cells expressing the antigen without cross-reactivity to non-specific proteins.

Eukaryotic cell lines

Policy information about cell lines

Cell line source(s)	HEK293T (ATCC CRT-3216) and HUVEC (ATCC CRL-1730) cells were purchased from ATCC.
Authentication	Cell lines were obtained directly from ATCC and were maintained frozen until use.
Mycoplasma contamination	cell lines were not tested for mycoplasma.
Commonly misidentified lines (See ICLAC register)	no commonly misidentified lines were used.

Animals and other organisms

Policy information about studies involving animals; ARRIVE guidelines recommended for reporting animal research

Laboratory animals	All mice used were between 4 and 8 weeks old and both male and female mice were used.<br>Msi2 reporter (Msi2eGFP/+) were kept on the B6 background<br>Actin-dsRed on the NOD/SCID background (NOD.Cg-PrkdcscidTg(CAG-DsRed*MST)1Nagy/KupwJ)<br>B6-CD45.2 and B6-CD45.1 (B6.SjL-PtprcaPepcb/BoyJ)<br>NOD/SCID (NOD.CB17-Prkdcscid/J)<br>NSG (NOD.Cg-PrkdcscidIL2rgth1Wji/SzJ)<br>Sdc1 KO mice (Sdc1-/-) were kept on the B6 background
Wild animals	the study does not involve wild animals.
Field-collected samples	the study does not involve field-collected samples.
Ethics oversight	All animal experiments were performed according to protocols approved by the University of California San Diego Institutional Animal Care and Use Committee.

Note that full information on the approval of the study protocol must also be provided in the manuscript.

Flow Cytometry

Plots

Confirm that:

- [x] The axis labels state the marker and fluorochrome used (e.g. CD4-FITC).
- [x] The axis scales are clearly visible. Include numbers along axes only for bottom left plot of group (a 'group' is an analysis of identical markers).
- [x] All plots are contour plots with outliers or pseudocolor plots.
- [x] A numerical value for number of cells or percentage (with statistics) is provided.

Methodology

Sample preparation	Bone marrow was recovered from femurs, tibias, and pelvis. Bones were crushed using a mortar and pestle, cells were suspended in Hanks' balanced salt solution (HBSS) (Gibco, Life Technologies) containing 5% (vol/vol) fetal bovine serum and 2 mM EDTA and filtered using a 70um filter. Red blood cells were lysed using RBC Lysis Buffer (eBioscience) and ckit cells were enriched by positive selection using magnetic beads (Miltenyi Biotec) on a AutoMACS Pro (Miltenyi Biotec) before staining for lineage markers. All antibodies were purchased from BD Pharmingen, eBioscience or BioLegend.<br><br>The high throughput surface antibody screen was performed per manufacturer's instruction (BD Lyoplate). Briefly, near terminal Msi2 reporter bcCML mice were sacrificed and the spleen was dissociated to produce a single cell suspensions. 500,000 cells were added to each well of a 96 well U-bottom plate. Cells were subsequently incubated with primary, biotin conjugated secondary, and streptavidin conjugated fluorescent tertiary antibodies while washing twice between antibody steps.<br><br>Analysis of leukemic mice was similarly done. Briefly, cells spleens and/or bone marrow from sick mice were prepared as described above to generate single cell supsensions that were then incubated with FACs antibodies.<br><br>See Supplementary Table 1 for details of all antibodies used.
Instrument	The high throughput antibody screen was performed using a BD FACSCANTO II equipped with a high throughput sampler.<br>All other analysis and cell sorting was carried out on a FACSAria III (BD Biosciences).
Software	FACSDiva version 6.1.3 (BD Biosciences) was used to collect data and data analysis was done using FlowJo version 10.5.3 (Tree Star Inc.).

Cell population abundance	The purity of post-sort KLS from B6 wild type and Sdc1-/- mice was higher that 95% as determined by flow cytometry.<br>The purity of post-sort transduced KLS cells was greater that 98% as determined by flow cytometry.
Gating strategy	KLS sort: morphology (FSC/SSC) -> live cells (PI-) -> lineage negative (lin-) -> ckit + & Sca1 + (ckit/Sca1).<br>BCR-ABL & NUP98-HOXA9 transduced KLS: morphology (FSC/SSC) -> live cells (PI-) -> NGFR+ (BCR-ABL vector) & CD2+ (NUP98-HOXA9 vector).<br>Primary bcCML: morphology (FSC/SSC) -> live cells (PI-) -> NGFR+ (BCR-ABL vector) & CD2+ (NUP98-HOXA9 vector) -> lineage negative (lin-).<br>shRNA transduced bcCML: morphology (FSC/SSC) -> live cells (PI-) -> GFP+ (shRNA vector).<br>Boundaries between "positive" and "negative" populations were defined using a fluorescence minus one sample.<br>See Supplementary Table 1 for antibody panels used. See Fig. S6 for gating examples.

☒ Tick this box to confirm that a figure exemplifying the gating strategy is provided in the Supplementary Information.
